# A Printed Hydrogel Hybrid Electronic System With Thermoresponsive Adhesion for Neurophysiological Monitoring

**DOI:** 10.1002/advs.76396

**Published:** 2026-06-29

**Authors:** Bo Pang, Ganguang Yang, Jiacheng Wen, Hangyu Gong, Caixin Gong, Yuqi Qiu, Zhixin Wang, Qingyang Zheng, Sen Zhou, Tianzhao Bu, Jia Tian, Zhouping Yin, Yutian Liu, Hao Wu

**Affiliations:** ^1^ Flexible Electronics Research Center, State Key Laboratory of Intelligent Manufacturing Equipment and Technology, School of Mechanical Science and Engineering Huazhong University of Science and Technology Wuhan China; ^2^ Department of Hand Surgery, Union Hospital, Tongji Medical College Huazhong University of Science and Technology Wuhan Hubei China; ^3^ School of Integrated Circuits Huazhong University of Science and Technology Wuhan Hubei China

**Keywords:** direct printing, neurophysiological monitoring, soft hybrid electronics, thermoresponsive hydrogel interfaces

## Abstract

Neurological disorders severely impair the daily life of patients, necessitating precise assessments of neural conduction pathway damage to guide effective diagnosis and treatment. However, current clinical neurodiagnostic equipment is expensive, bulky, and restricted to hospital settings. Furthermore, the rigid nature and low adhesion of conventional electrodes lead to the acquisition of low‐quality neurophysiological signals. Here, we develop a soft hybrid electronic system assembled with printed responsive hydrogel electrodes. By synthesizing thermoresponsive hydrogel microsphere inks, we achieve rapid direct‐printed patterning of interface layers. Based on a thermally triggered hydration‐dehydration strategy of hydrogel microspheres, the hydrogel electrodes demonstrate customizable adhesion, enabling intimate integration with skin for high‐fidelity signal acquisition and gentle detachment after monitoring. Through hydrogel interface modification, on‐skin electrodes also show excellent charge injection capability for stimulation and improved signal‐to‐noise ratio (SNR). The integration of hydrogel patches with the hybrid electronic system enables high‐frequency and high‐fidelity acquisition of subtle neural conduction signals. We further apply this system to assess injuries to the median and ulnar nerves in clinical cases, realizing accurate diagnosis of neural impairment while ensuring customized adhesion regulation. This user‐friendly system holds great promise as a low‐cost and portable diagnostic platform for telemedicine, home‐based care, and clinical settings.

## Graphical abstract

This work develops a soft hybrid electronic system with printed thermoresponsive hydrogel electrodes, which enables high‐fidelity neural signal acquisition and stimulation. The system precisely assesses median and ulnar nerve injuries in clinical cases, realizing accurate diagnosis of neural impairment while ensuring customized adhesion regulation. This low‐cost, portable platform expands neurodiagnostic capabilities to telemedicine, home‐based care, and clinical settings.

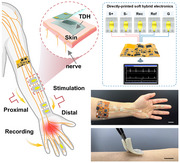

## 1 Introduction

Neurological disorders of the central, peripheral, and autonomic nervous systems have imposed substantial burdens on global health over the past few decades [1, 2, 3]. Some 22.6 million patients worldwide require medical care annually due to neurological disorders, with a constantly increasing trend [4, 5]. There is an urgent need to ensure early detection and timely intervention of these diseases [6]. Currently, neurophysiological monitoring is widely employed in clinical practice to evaluate the integrity of neural conduction pathways [7, 8, 9]. Specifically, rigid metal electrodes deliver stimulating pulsed currents that propagate along nerve fibers, triggering neuronal action potentials and ultimately eliciting muscle activation. These signals are captured by recording electrodes at the target site [10, 11, 12]. Nerve injury is generally characterized by abnormalities of electrophysiological signals in amplitude, latency, or conduction velocity when compared with normal thresholds [13, 14, 15]. Therefore, neurophysiological monitoring requires coordinated use of stimulating and recording electrodes combined with sophisticated hardware systems to acquire accurate signals and evaluate nerve integrity [16, 17, 18]. However, traditional commercial systems for electromyography and evoked potential recording are typically bulky, wire‐connected, and confined to specialized clinical settings [19]. Due to high cost, commercial systems severely impose a substantial financial burden on patients who require long‐term monitoring [20]. The development of portable electrophysiological monitoring systems remains challenging, as the rapid conduction velocity of neural signals demands high sampling rates, while the low signal amplitudes require high‐fidelity acquisition to minimize noise interference. Furthermore, owing to the different locations of nerve injury, the amplitude of monitored signals can range from microvolts to millivolts. Conventional rigid electrodes characterized by high modulus and low adhesion present considerable challenges in forming conformal contacts with skin for capturing high‐fidelity potential signals [21, 22]. Additionally, the unadjustable adhesion properties cannot meet customized interface adhesion requirements of clinical cases (e.g., loose, fragile, and postoperative skin), further compromising signal quality and patient wear comfort [23]. As a result, existing electrodes/systems still have difficulty in enabling high‐performance stimulation and recording for diagnosis.

In recent years, advances in flexible sensing systems have prompted numerous efforts to improve device/skin interfaces and develop a range of on‐skin electronic systems using stretchable materials, aiming to enhance signal fidelity [24, 25, 26]. However, existing flexible systems still exhibit low adhesion, which hinders them from forming and maintaining conformal contact with skin. It is highlighted that hydrogels are emerging as promising soft materials for on‐skin electronics, due to their remarkable adhesion, low modulus, and biocompatibility [27, 28, 29]. Specifically, self‐adhesive hydrogel interfaces enable robust bonding with the skin, enhancing both signal integrity and charge injection efficiency. Nevertheless, clinical scenarios impose conflicting demands on the electrode/skin interface: superior adhesion throughout monitoring while gentle detachment upon completion [30]. While several responsive hydrogels with tunable adhesion have been reported, their fabrication processes rely on conventional mold pouring methods, with limitations including time‐consuming processes and inevitable interface damage during demolding that significantly impair manufacturing efficiency [31, 32, 33, 34, 35, 36]. Overall, integrated electrode systems capable of tunable adhesion, convenient use, long‐term high‐fidelity electrical stimulation, and signal acquisition remain to be developed for neurological diagnostics.

Here, we report a soft hybrid electronic system integrated with thermoresponsive directly‐printed hydrogel (TDH) electrodes capable of achieving simultaneous electrical stimulation and high‐fidelity signal acquisition for neurophysiological monitoring. We synthesize thermoresponsive hydrogel microspheres (THMs) to modify the rheological properties of the hydrogel ink, enabling precise, on‐demand control over the viscosity. By using the direct‐writing platforms loaded with inks, the hydrogel interface layer is patterned onto the soft substrate to fabricate the hydrogel electrode while preserving structural integrity. To improve the initial adhesion of hydrogels, we conjugate dopamine (DA) onto sodium carboxymethylcellulose (CMC) to yield carboxymethylcellulose‐dopamine hydrochloride (CMC‐DA) chains for establishing robust coupling with carboxyl and amino groups on the skin surface. It is noted that based on a hydration‐dehydration strategy of hydrogel microspheres triggered by heat, the hydrogel interface achieves wide‐range adhesion regulation, facilitating rapid tight coupling with skin for high‐quality signal monitoring and gentle detachment without causing skin injury. Electrical property evaluations demonstrate that the TDH electrodes exhibit superior charge injection capability (CIC), higher charge storage capacity (CSC), and lower contact impedance compared with commercial electrodes. Moreover, under wet and vibratory conditions, the TDH electrodes maintain stable adhesion to the skin and collect precise surface electromyography (sEMG) signals. Animal experiments further confirm significantly enhanced amplitude in characteristic neurophysiological peaks, demonstrating the superior ability to acquire higher‐fidelity somatosensory evoked potential (SSEP) signals. By integrating the TDH electrodes, the hybrid electronic system achieves a broad stimulation current range of 8–50 mA and 16 kHz signal acquisition across microvolt to millivolt levels. Furthermore, the system is evaluated for the clinical diagnosis of both motor and sensory branches of the median and ulnar nerves. Analysis of amplitude, latency, and conduction velocity in patient electrophysiological waveforms enables accurate assessment of nerve injury severity.

## 2 Results and Discussion

### 2.1 Design Overview and System Architecture

**FIGURE 1:**
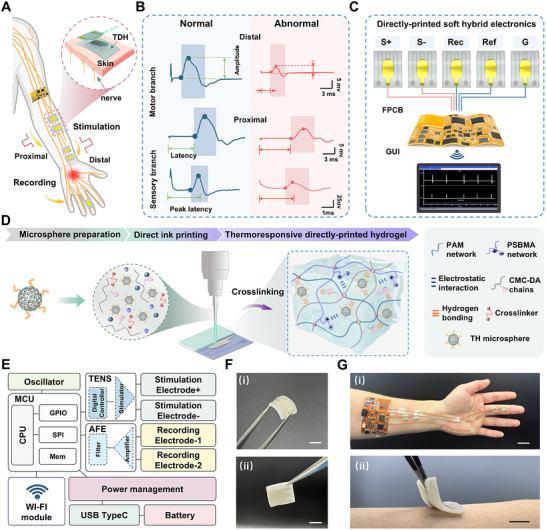
Design architecture of the thermosensitive directly‐printed hydrogel hybrid electronic system for neurophysiological diagnosis. (A) Schematic illustration of thermosensitive directly‐printed hydrogel (TDH) electrodes for diagnosis of the peripheral nervous system. (B) Analysis of electrophysiological signals in subjects with normal and abnormal neurological function. (C) Schematic architecture of the hardware system. S+, stimulating anode; S−, stimulating cathode; Rec, recording electrode; Ref, reference electrode; G, ground; GUI, graphical user interface. (D) Schematic illustration of the compositions and preparation of TDH. (E) Functional diagram of the electronic system. TENS, transcutaneous electrical nerve stimulation; MCU, microcontroller unit; GPIO, general‐purpose input/output port; SPI, serial peripheral interface; Mem, memory; AFE, analog front end. (F) Photographs of the TDH electrode from front (i) and back (ii) views. (G) Optical images of the hybrid electronic system conformally attached to the skin (i) and TDH electrode peeled from the skin (ii). Scale bar, 10 mm (F), 20 mm (G–i), 5 mm (G–ii).

The system consists of three components: TDH electrodes for high‐fidelity biosensing, a flexible printed circuit board (FPCB) for signal acquisition and transmission, and a dedicated graphical user interface (GUI) for real‐time data visualization. As shown in Figure 1A, when the hydrogel electrodes are affixed to the skin to stimulate the underlying peripheral nerves and record the potential signals, the flexible system enables non‐invasive diagnosis of peripheral nerve function. For functional assessment, we position the TDH electrodes on specific sensory and motor branches. The electrode records potentials from the nerves following distal and proximal stimulation. Amplitude, latency, and conduction velocity serve as essential parameters for evaluating neural potentials. These parameters exhibit distinct threshold ranges for sensory and motor branches across different regions. Abnormal neurological function typically manifests as reduced amplitude, prolonged latency, and decreased conduction velocity. Comparing the measured values with normal thresholds provides the basis for quantitative assessment of neurological dysfunction (Figure 1B). As shown in Figure 1C, the operational setup employs five TDH electrodes: two for electrical stimulation (designated S+ and S−) and three for signal acquisition (Rec, Ref, and G). During operation, the acquisition terminals are activated concurrently with the delivery of electrical stimulation to the peripheral nerves. The FPCB module subsequently captures the evoked potential signals, wirelessly transmitted to a host computer via a Wi‐Fi protocol, and the GUI displays the waveform for real‐time monitoring. To fabricate the TDH interface layer, thermoresponsive microsphere‐based inks are directly printed onto the soft electrode and subsequently undergo ultraviolet‐initiated polymerization to form stable hydrogel networks. The TDH layer comprises polyacrylamide (PAM), CMC‐DA chains, and poly(sulfobetaine methacrylate) (PSBMA), synergistically cross‐linked through both physical entanglement and chemical bonds. The networks are further decorated with porous THMs, which are assembled via hydrogen bonding and electrostatic interactions (Figure 1D). To prepare porous THMs, we synthesize thermoresponsive N‐isopropylacrylamide (NIPAM)/polyaspartic acid (PASP) microspheres (NPMs), followed by liquid nitrogen treatment (LT) to fabricate porous structures. As shown in Figure 1E and Note S1, signal acquisition is controlled via the MCU, which manages data transmission and reception. The reliable capture of high‐fidelity electrophysiological signals is technically challenging due to their high conduction velocity and low amplitude, particularly in sensory pathways. The analog front‐end (AFE) for signal acquisition incorporates programmable amplifiers and filters, operating at a high sampling rate of 16 kHz with a common‐mode rejection ratio (CMRR) of −110 dB, ensuring high‐frequency acquisition of subtle neural signals. For stimulation, a dedicated transcutaneous electrical nerve stimulation (TENS) chip delivers precise electrical pulses to the target nerves, supporting programmable current output across ten levels (8–50 mA).

Polydimethylsiloxane (PDMS) serves as the stretchable substrate for the TDH electrodes, providing dual functionality for both electrical stimulation and signal recording. Electrically conductive composites (ECCs) are patterned on the upper and lower PDMS surfaces to function as the electrode and heater, respectively (Figure 1F). The ECC is composed of iodination‐photoreduction‐modified silver flakes (75 wt%) dispersed in a polymer matrix (i.e., PDMS), with a thickness of ∼50 µm. It serves as an essential material for the fabrication of stretchable electrodes, ensuring long‐term stable conductivity, low impedance, and a high signal‐to‐noise ratio (SNR) [37]. The TDH interface layer is fabricated onto the surface of the ECC electrode via direct printing. The catechol groups of CMC‐DA chains in TDH establish robust coupling with carboxyl and amino groups on the skin surface through multimodal physical and chemical interactions, enabling strong coupling of hydrogel/skin interfaces. The porous hydrogel microsphere structures exhibit thermoresponsive properties and possess a strong capacity to absorb water molecules. At room temperature, the polymer networks in microspheres trap the water molecules through hydrogen bonding, presenting loose structures. The TDH electrodes can establish intimate integration with the skin (Figure 1G i). After completion of monitoring, the hydrogel interface layer is heated to initiate a thermal phase transition, prompting rapid dehydration of the swollen microspheres. As the temperature rises, the formation of hydration layers at the hydrogel/skin interface leads to the dissociation of adhesive bonds and a substantial reduction in interfacial adhesion. The heat‐triggered adhesion mechanism enables the benign detachment between electrode and skin (Figure 1G ii).

### 2.2 Synthesis and Characterizations of Tunable‐Adhesion Hydrogel Microspheres

**FIGURE 2:**
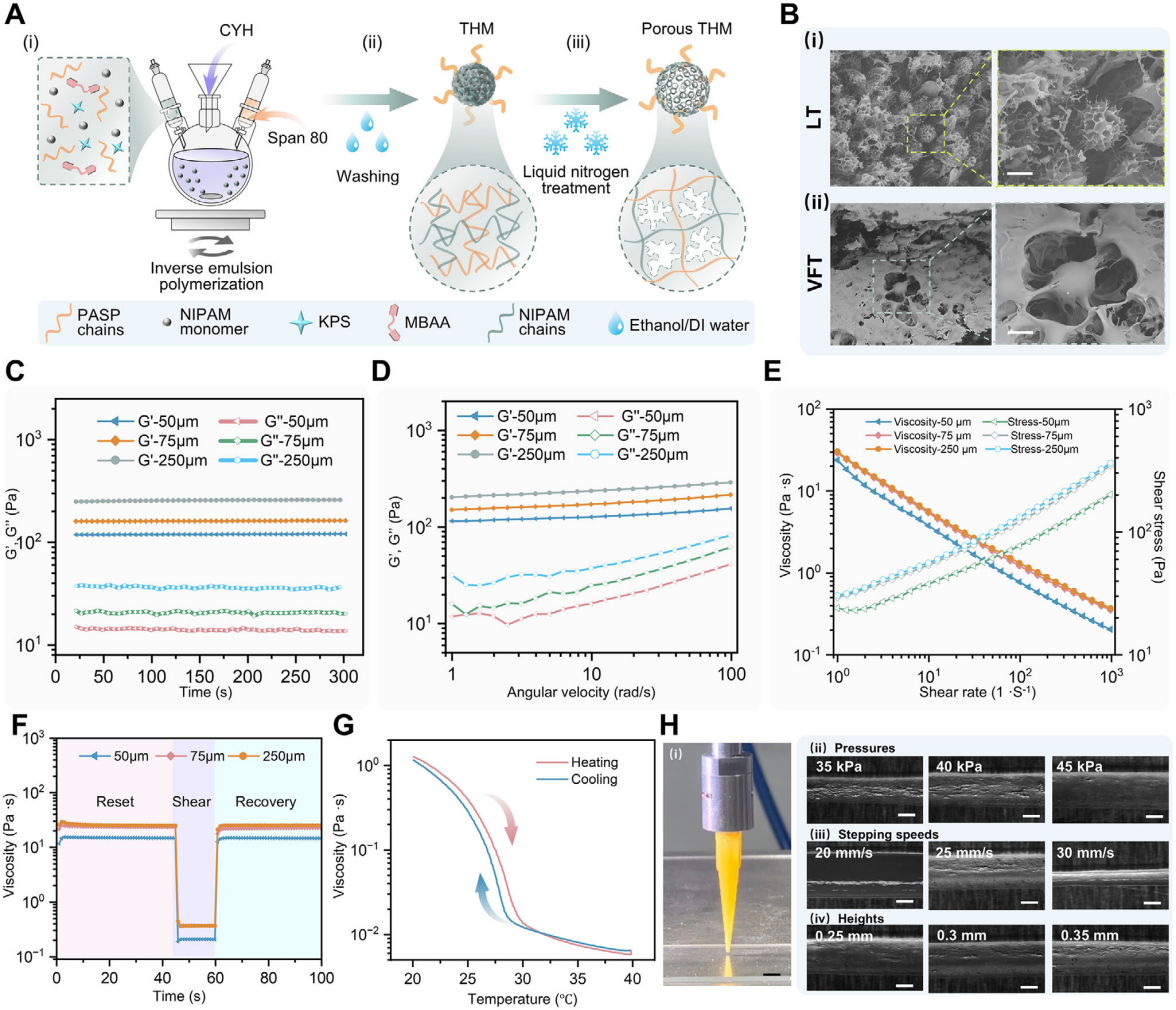
Synthesis and characterization of the thermoresponsive PNPMs. (A) Schematic illustrations of the synthesis process of thermoresponsive hydrogel microspheres (i.e., PNPMs). (B) SEM images of PNPMs processed by LT (i) and VFT (ii), respectively. (C and D) Storage (G') and loss (G”) modulus changes over time (C) and angular velocity (D) of hydrogel inks. (E) Viscosity‐shear rate and shear stress‐shear rate curves of the microspheres. (F) Three‐interval thixotropy test (3ITT) curves of the abovementioned microsphere sizes. (G) Viscosity changes of PNPMs during thermal cycling (20°C–40°C–20°C). (H) Optical images of the printing process (i) and microscope images under different pressures (ii), stepping speeds (iii), and nozzle heights (iv). Scale bars, 80 µm (left), 20 µm (right) (B), 0.8 mm (F).

To achieve rapid fabrication of thermoresponsive hydrogel interface layers, we developed printable hydrogel inks. Particularly, porous hydrogel microspheres serve as the core components that endow the ink with both printability and temperature‐responsive properties. As shown in Figure 2Ai–ii and Figure S1A–F, THMs were first synthesized via inverse emulsion polymerization using NIPAM monomers and PASP chains. After centrifugal washing, the THMs were subjected to liquid nitrogen treatment (LT) followed by lyophilization to produce porous microstructures, thereby enhancing their water absorption capability (Figure 2A iii and Figure S1G–I). To verify the successful synthesis of porous THMs, we compared scanning electron microscopy (SEM) images of hydrogel microspheres prepared with LT and vacuum‐freeze‐drying treatment (VFT) processes. As shown in Figure 2B, THMs (LT) exhibit larger diameters and distinct porous structures, whereas hydrogel microspheres without LT appear smaller with smooth surfaces. This difference is attributed to the fact that the LT process instantaneously freezes the water within the swollen thermosensitive hydrogel microspheres, forming ice crystals that maintain their size and morphology throughout lyophilization. Subsequent sublimation of the ice crystals yields a porous structure with increased surface area for water interaction, significantly improving hydration capacity. In contrast, hydrogel microspheres without LT undergo gradual dehydration, resulting in shrinkage and smooth spherical morphologies.

To further evaluate the rheological performance of inks prepared by using porous microspheres with different diameters, we first sieved the synthesized hydrogel microspheres through mesh filters of 250, 75, and 50 µm, obtaining three size fractions of THMs. These microspheres were then equally dispersed in deionized (DI) water and allowed to fully swell at room temperature (20°C). Subsequent oscillatory shear tests were conducted to evaluate the rheological behaviors of the three ink formulations at 20°C. As shown in Figure 2C,D, THM inks (250 µm) present the highest initial storage modulus (G'_time_: 246.3 Pa, G'_ω_: 287.0 Pa) and loss modulus (G″_time_: 36.8 Pa, G″_ω_: 80.9 Pa) under both time‐ and frequency‐dependent tests, significantly exceeding the THM inks (75 µm) (G'_time_: 158.5 Pa, G'_ω_: 213.9 Pa; G″_time_: 21.1 Pa, G″_ω_: 60.7 Pa) and THM inks (50 µm) (G'_time_: 117.0 Pa, G'_ω_: 153.7 Pa; G″_time_: 14.7 Pa, G″_ω_: 40.7 Pa). When characterized at 40°C, all three inks show a marked reduction in both moduli, which is ascribed to the thermally induced conformational transition of PNIPAM chains (Figure S2). Specifically, at 20°C, the extended hydrophilic PNIPAM chains facilitate water absorption and swelling, with larger microspheres enabling denser packing in the ink matrix, thereby restricting flow. Upon heating, the disruption of hydrogen bonds between PNIPAM chains and water molecules triggers dehydration and hydrophobic aggregation, leading to water expulsion and enhanced fluidity. Shear rate‐dependent viscosity measurements demonstrate shear‐thinning behavior in all three inks, which facilitates the inks to ensure rapid and uniform extrusion during printing (Figure 2E). Furthermore, three‐interval thixotropy tests (3ITT) confirm rapid viscosity recovery of THM inks, indicating excellent structural stability of inks during the printing process (Figure 2F).

We further characterized the temperature‐dependent viscosity of microsphere‐based inks. As shown in Figure 2G, THM inks show the highest viscosity (1.266 Pa·s) at 20°C. Upon gradual heating to 40°C, the viscosity decreases significantly due to the thermal phase transition of microspheres and subsequent water expulsion, stabilizing at approximately 0.006 Pa·s. Notably, when cooled back to 20°C, the viscosity of inks recovers close to the initial value (∼1.148 Pa·s), proving the reversible thermoresponsive behavior of inks. To quantitatively evaluate the influence of printing parameters (including pressure, platform speed, and nozzle height) on printability, we recorded the printed ink trajectories under varying conditions (Figure S3). As shown in Figure 2H, Figures S4 and S5, by reducing the pressure, increasing the platform speed, and lowering the nozzle height, the line width of ink trajectories can be reduced, thereby achieving higher precision of printing. Notably, when the pressure, platform speed, and nozzle height are maintained at 35–45 kPa, 20–30 mm/s, and 0.25–0.35 mm, respectively, the printed ink exhibits uniform and continuous deposition, achieving a minimum line width of 760 µm (Note S2 and Movie S1). These findings further verify the outstanding printability of microsphere‐based hydrogel inks.

### 2.3 Adhesion Regulation Mechanism and Properties of Hydrogel Interfaces

**FIGURE 3:**
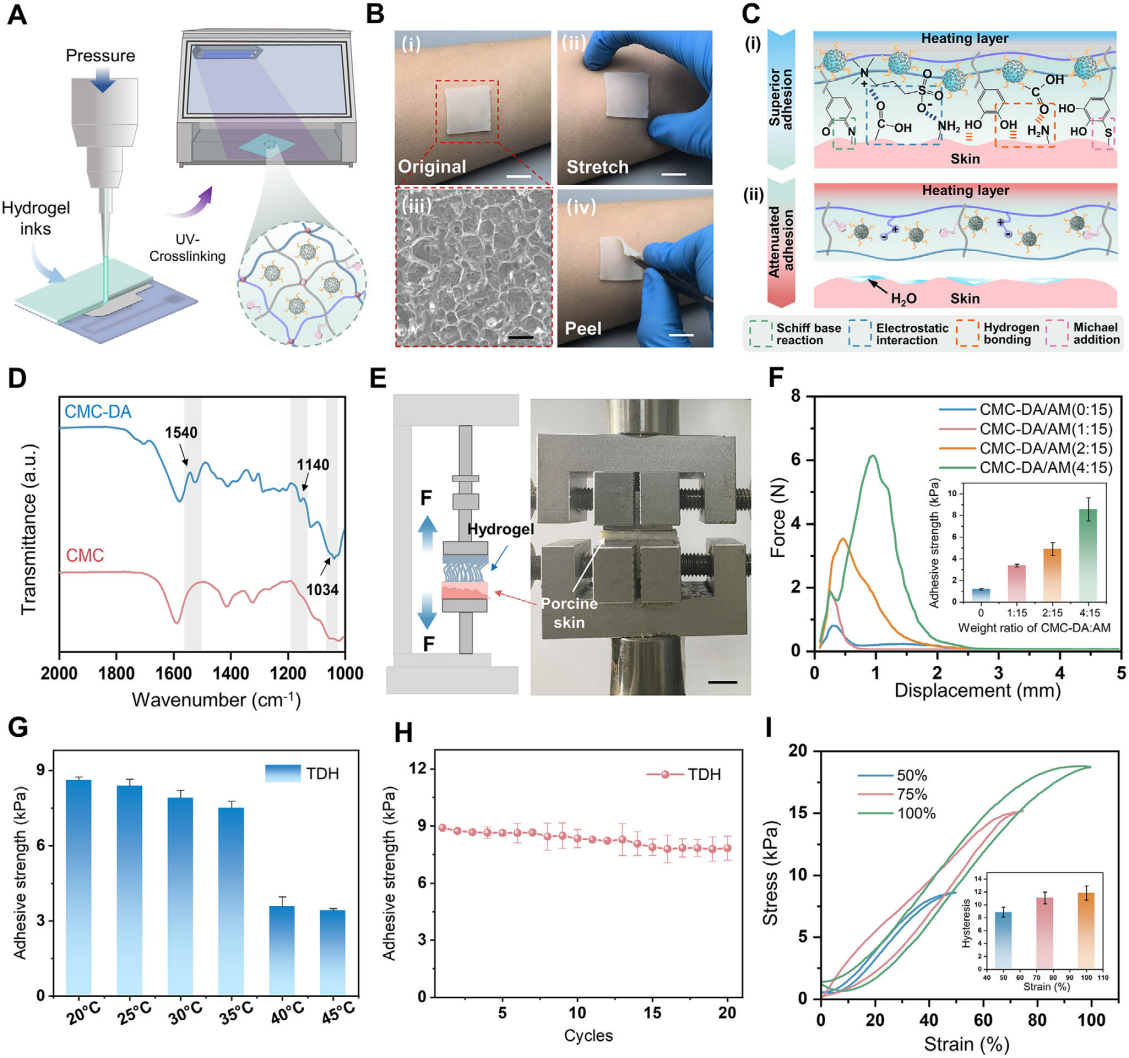
Mechanical properties of the hydrogel interface. (A) Schematic of the directly printing and crosslinking process for hydrogel inks. (B) Optical images of the TDH conformally attached to the skin (i), stretched (ii), peeled (iii), and SEM images of the thermosensitive directly‐printed microsphere‐based hydrogel (iv). (C) Adhesion regulation mechanism of the hydrogel/skin interface enabling intimate integration with skin (i) and gentle disassembly triggered by heat (ii). (D) FTIR‐ATR spectra of the CMC‐DA, CMC‐based hydrogel in the range of 1000–2000 cm^−1^. (E) Schematic of the tensile strength test (ASTM F2258) and a photograph of the TDH adhered to the pigskin. (F) Adhesive‐strength curves of TDH at different CMC‐DA concentrations. (G) Adhesive strength of the TDH at different temperatures. (H) Adhesive strength of TDH during 20 cycles of repeated peeling. (I) Stress–strain curves and hysteresis of the TDH under different loading‐unloading conditions from 0% to 50%, 75%, and 100% strain. Scale bars, 5 cm (C, (i), (ii), (iii)), 60 µm (C, (iv)), and 5 cm (E).

We formulated the hydrogel prepolymer solution by incorporating monomers and adhesive components into the microsphere‐based inks. It is highlighted that the microsphere inks remain nearly consistent rheological properties before/after the addition of these functional components, maintaining excellent printability. Based on this hydrogel prepolymer ink, we achieve rapidly patterned printing, followed by UV‐induced interfacial polymerization to obtain the thermoresponsive hydrogel interface layer (Figure 3A). The resulting TDHs exhibit remarkable softness, enabling conformal attachment to skin even under stretching while leaving no residue upon removal. Cross‐sectional SEM imaging reveals the porous network structures embedded with hydrogel microspheres (Figure 3B). It is noted that the incorporation of thermoresponsive microspheres endows TDHs with tunable adhesion, operating through the following thermally triggered mechanism (Figure 3C): at 20°C, the PNIPAM networks in microspheres maintain an expanded chain conformation due to hydrogen bonding between hydrophilic amide groups and water molecules. Due to the decoration of CMC‐DA, zwitterionic moieties, and carboxyl groups, TDHs establish robust adhesion to the skin through Michael addition, Schiff base formation, electrostatic interactions, and hydrogen bonding (Figure 3Ci). Upon heating, the hydrogen bonds between PNIPAM chains and water molecules dissociated, prompting chain collapse into hydrophobic aggregates with concomitant water expulsion. The formed hydration layer disrupts the hydrogen bonds and electrostatic couplings of hydrogel/skin interfaces, substantially weakening the adhesion of hydrogels, thereby achieving gentle detachment (Figure 3Cii). Importantly, the modification of hydrogel networks with CMC‐DA and zwitterionic chains enhanced the initial adhesion strength, thereby achieving a broader range of adhesion tunability. We improve the initial adhesion of hydrogel layers by modifying CMC‐DA and zwitterionic chains to achieve wider‐range adhesion regulation.

To verify the successful grafting of catechol groups on the CMC chains, we systematically characterized CMC‐DA using spectroscopic techniques. Fourier‐transform infrared spectroscopy with attenuated total reflection (FTIR‐ATR) analysis reveals new characteristic absorption peaks at 1540, 1140, and 1034 cm^−1^ in CMC‐DA compared to pristine CMC, corresponding to the benzene ring C═C vibration and phenolic O─H vibrations of catechol groups (Figure 3D). Furthermore, ultraviolet–visible (UV–vis) and proton nuclear magnetic resonance (^1^H NMR) spectra also exhibit the distinct absorption peaks of CMC‐DA at 280 nm and 6.78 ppm, respectively, confirming successful DA modification (Figure S6). To validate the incorporation of zwitterionic networks, we performed X‐ray photoelectron spectroscopy (XPS) comparative analysis on TDH interface layers with/without SBMA. As shown in Figure S7, the emergence of a sulfur‐specific peak at 164.7 eV, which is attributed to the S2p of sulfonate groups (SO_3_) in PSBMA, proves the presence of zwitterionic chains in hydrogel networks.

As shown in Figure 3E, standardized tensile tests (ASTM F2258) were performed on TDH interface layers decorated with different weight ratios of CMC‐DA: AM. The results show a remarkable increase in adhesive strength from 1.2 to 8.6 kPa as the CMC‐DA ratio increased from 0 to 4:15, which is ascribed to the enhanced chemical and physical interactions facilitated by the additional catechol groups from CMC‐DA (Figure 3F). We also characterized the adhesion properties of hydrogel interface layers with different compositions (Figure S8). The results show that the grafting of DA onto CMC effectively enhances the hydrogel adhesion, whereas the modification with PNIPAM microspheres endows hydrogel networks with thermal responsiveness and printability rather than adhesion improvement. TDHs also present universal adhesiveness, forming robust coupling with various porcine tissues (e.g., heart, stomach, and liver) (Figure S9 and Note S3). Based on comprehensive considerations of skin tolerance and hydrogel adhesive stability, we regulate the proportion of hydrophilic components within hydrogel networks to achieve the lower critical solution temperature (LCST) exceeding 36°C (Figure S10). At 20°C, the hydrogel demonstrates the adhesive strength of approximately 8.6 kPa. Upon further heating to 40°C, the hydrogel interface layer undergoes a thermal phase transition, resulting in a significant reduction of adhesive strength to 3.6 kPa, which enables gentle detachment from the skin (Figure 3G). Moreover, TDH interface layers show superior adhesive strength (>7 kPa) during 5‐day tests and wet‐skin tests (Figure S11). Besides, the adhesive strength of hydrogels slightly decreases and remains at 7.8 kPa after twenty cycles, confirming the adhesion stability of hydrogel interface layers (Figure 3H).

We further evaluated the mechanical properties of TDH interface layers through tensile strain tests. As shown in Figure S12, TDHs demonstrate exceptional stretchability (∼250%) along with the ultralow modulus of 1.3 kPa, which is dramatically lower than that of conventional flexible materials such as polydimethylsiloxane (693.9 kPa) and Ecoflex (58.6 kPa), confirming the superior mechanical compliance of hydrogels. Loading‐unloading tests were conducted under varying strains (0%–100%) and 10 consecutive 50% strain cycles. Figure 3I and Figure S13A show that TDH maintains consistent low hysteresis with values below 15%. This characteristic arises from the synergistic effect of multiple interactions within hydrogel networks, including covalent crosslinking, hydrogen bonding, and electrostatic coupling, which ensures stable interfacial performances during cyclic mechanical deformation for achieving reliable skin/device integration. We also measured the weight change of hydrogel interface layers before/after 24‐h storage at 25°C and 56% humidity. As shown in Figure S13B, the hydrogel layer still retains 79% of its weight after 24 h, further confirming its stability.

### 2.4 Electrical Performances of Printed Hydrogel Electrodes

**FIGURE 4:**
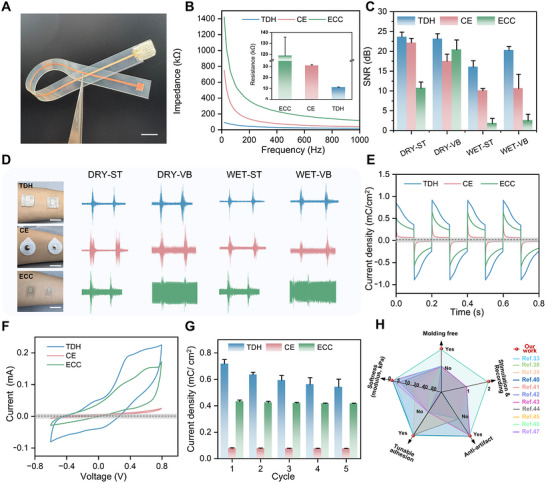
Electrical properties of the TDH electrode. (A) Photograph of the TDH electrode. (B) Comparisons of contact impedance between TDH electrode, commercial electrode (CE), and ECC electrode. Inset shows the corresponding contact impedance of each electrode at 1 kHz. (C) SNR values of the abovementioned electrodes under static (ST, dry/wet skin) and vibratory (VB, dry/wet skin) conditions. (D) Comparisons of surface electromyography (sEMG) signals recorded by the abovementioned electrodes. (E and F) Charge injection curves (E) and cyclic voltammetry curves (F) of the three different electrodes. (G) Charge storage capacity (CSC) of different electrodes under various cycling conditions. (H) Comparisons of the TDH electrode and previously reported flexible electrodes in terms of softness, tunable adhesion, molding‐free, and anti‐artifact properties. Data in (B), (C), and (G) are presented as means ± SD, *n* = 3. Scale bars, 10 mm (A) and 10 mm (D).

The TDH layer was assembled onto the PDMS‐ECC substrate via direct printing to fabricate the electrode (Figure 4A). Specifically, the PDMS surface was treated with benzophenone to activate grafting sites after fabricating the ECC electrode and heating layer. This treatment facilitates covalent cross‐linking between the hydrogel and PDMS, significantly enhancing interfacial bonding strength (Figure S14). Heat‐triggered detachment is preferred owing to its fast thermal response, controllable temperature, and tunable adhesion, rendering it suitable for various skin types in clinical settings. The TDH electrodes show stable adhesion regulation properties and maintain structural integrity during five adhesion‐debonding tests triggered by heat (Figure S15). Infrared thermal images of the heater demonstrate that the device can achieve uniform heating within 10 s, ensuring full detachment across the patch (Figure S16). The TDH electrode can also exhibit a stretchability of 100% and remains stable after 200 cycles of stretching and bending (Figure S17). We also characterized the contact impedance of the TDH electrode, the commercial electrode Ag/AgCl (CE), and the ECC electrode on skin (Figure 4B). The TDH electrodes demonstrate significantly lower impedance of 96.42 kΩ (at 20 Hz) and 10.87 kΩ (at 1 kHz), compared with CE (20 Hz: 721.11 kΩ, 1 kHz: 30.36 kΩ) and ECC electrode (20 Hz: 1359.41 kΩ, 1 kHz: 117.21 kΩ), respectively. These results confirm the superior recording performance of TDH electrodes. As shown in Figure 4C, we employed the above‐mentioned three types of electrodes to measure sEMG signals under different test scenarios (i.e., static state (dry skin), vibratory (dry skin), static state (wet skin), and vibratory (wet skin)). The TDH electrode consistently demonstrates a high SNR (>23 dB) under dry, static conditions and retains stable performances under complex environmental variations (e.g., wet skin and vibratory states). Moreover, the SNR of TDH electrodes significantly surpasses that of both commercial and ECC electrodes (Note S4). The TDH electrodes retain strong adhesion under wet conditions, whereas commercial and ECC electrodes are highly susceptible to such environmental challenges, leading to adhesion loss and significant degradation of signal fidelity. Noticeably, under vibratory conditions, commercial electrodes and ECC patches exhibit significant attenuation in SNR or even fail to capture sEMG signals. In contrast, the TDH layer effectively mitigates motion artifacts, still collecting high‐quality electrophysiological (EP) signals (Figure 4D). Furthermore, the SNR and contact impedance remain unchanged across the tested temperature range, owing to the conformal contact at the interface (Figure S18).

The stimulation performance of the TDH electrodes was quantitatively evaluated based on the calculated CIC and CSC (Note S5). As shown in Figure 4E, the TDH electrodes exhibit the highest CIC (0.107 mC/cm^2^), compared with commercial electrodes (0.011 mC/cm^2^) and ECC electrodes (0.061 mC/cm^2^). Furthermore, the TDH electrodes demonstrate exceptional stability, with CIC values remaining consistent across four consecutive cycles (0.107, 0.111, 0.111, and 0.111 mC/cm^2^), confirming reliable charge injection performance (Figure S19). As shown in Figure 4F,G, the CSC values for TDH, CE, and ECC electrodes are 0.611, 0.075, and 0.451 mC/cm^2^, respectively. The TDH electrodes maintain stable CSC in five cycles, attributed to the superior effective surface area of the hydrogel design, which enhances charge‐discharge ability. As shown in Figure 4H and Table S1, the TDH electrode exhibits superior characteristics (i.e., softness, tunable adhesion, rapid printability, and anti‐artifact properties), exhibiting the highest overall performance when compared with reported systems [33, 38, 39, 40, 41, 42, 43, 44, 45, 46, 47].

### 2.5 In Vitro Biocompatibility and Animal Tests

**FIGURE 5:**
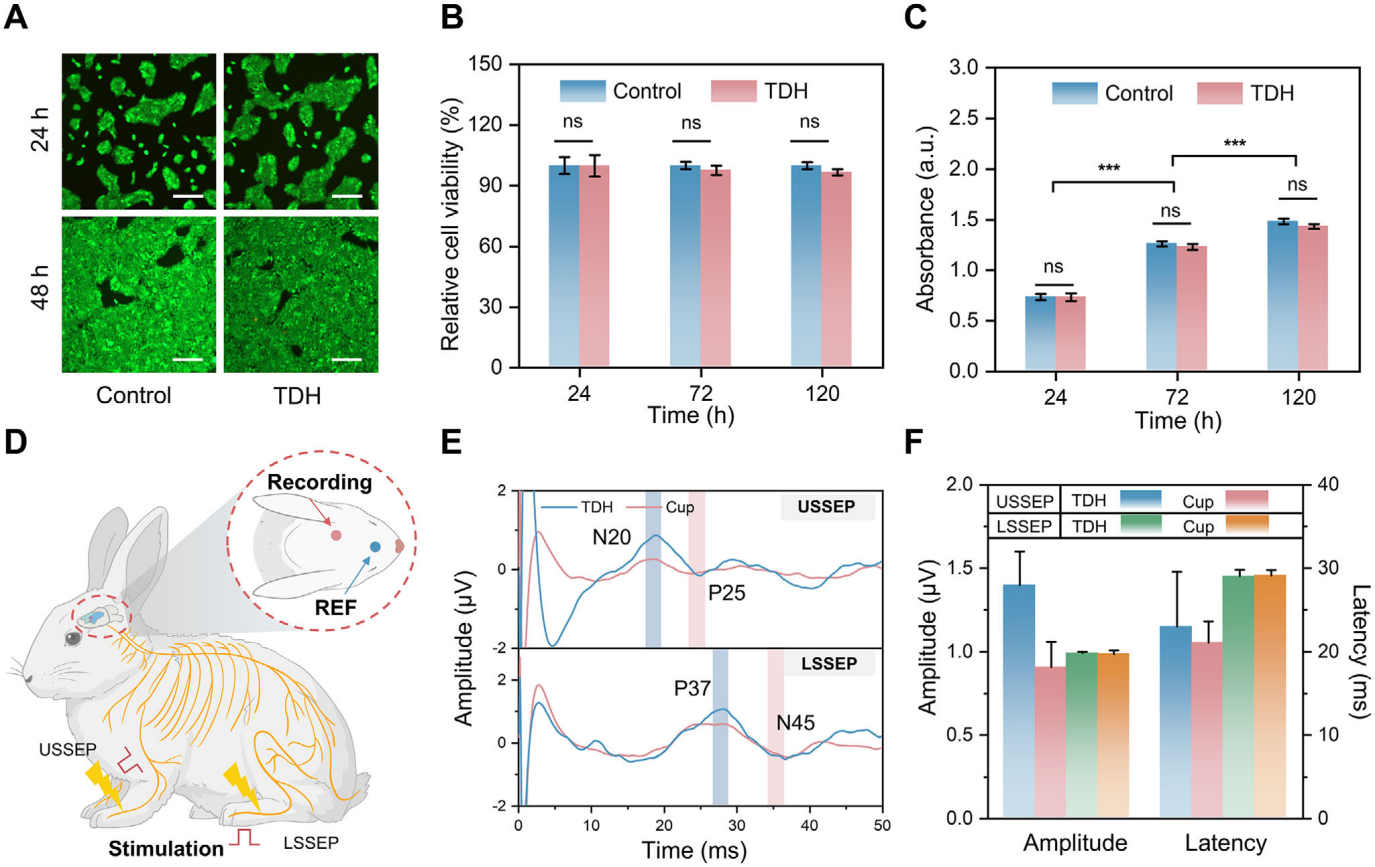
Biocompatibility evaluation of TDH electrodes and animal tests of SSEP. (A) Fluorescent microscopy images of human immortalized keratinocyte (HaCaT) cultured with TDH (test group) and PDMS (control group), stained with calcein‐AM (live cells, green) and propidium iodide (PI, dead cells, red). (B and C) Relative cell proliferation (Rp, B) and viability (Rv, C) of HaCaTs cultured in the TDH‐based medium calculated by CCK‐8 assay. (D) Schematic illustration of USSEP and LSSEP. (E) Comparisons of SSEP waveform signals recorded using TDH electrodes and CE electrodes. ‘N20’ and ‘N45’ represent the peak of negative potential at a latency of approximately 20 ms and 45 ms, respectively. ‘P25’ and ‘P37’ represent the peak of positive potential at a latency of approximately 25 ms and 37 ms, respectively. (F) Amplitude and latency comparisons of the SSEP signals collected by TDH and Cup electrodes. *P* values are determined by two‐sided Student's *t*‐test between two groups and ANOVA with Least Significant Difference (LSD) post hoc test between multiple groups, respectively; ^*^ *p* < 0.05, ^**^ *p* < 0.01, and ^***^ *p* < 0.001; ns, not significant. Data in (B) and (C) are presented as means ± SD, *n* = 3. Scale bars, 100 µm (A).

Given that the TDH interface is directly adhered to human skin, assessing its biocompatibility is important. We conducted animal experiments to evaluate the cytotoxicity of the TDH electrodes by using human immortalized keratinocytes (HaCaTs). In vitro cytotoxicity evaluation shows nearly identical proliferation rates for HaCaT cells in the control and hydrogel groups, with no significant apoptosis or necrosis observed throughout the study period (Figure 5A). Additionally, we quantitatively analyzed the relative cell viability (*Rv*) and proliferation rate (*Rp*) of HaCaTs and fibroblasts incubated with the hydrogels. The hydrogel groups exhibit high biocompatibility, with *Rv* > 96% and *Rp* > 1.4 after 5 days of culture, showing no statistically significant difference when compared with control groups (Figure 5B,C). We further evaluated the biocompatibility of flexible electrodes by attaching them to the forearms of human subjects and performing two separate tests: 20 repeated peeling cycles and a 60‐min continuous adhesion test. Visual inspection of the skin contact areas reveals no signs of allergic reaction or erythema, confirming the excellent cutaneous biocompatibility of the electrodes (Figure S20). Meanwhile, long‐term skin stability tests show no significant changes in impedance or SNR compared with the initial state after 48 h of wearing, and no skin irritation was observed (Figure S21).

We further investigated the clinical applicability of the TDH electrodes by assessing their capability to monitor functional states of the central nervous system in rabbit models through SSEP recordings [48, 49]. TDH electrodes and conventional cup electrodes were used in animal experiments for parallel monitoring of electrophysiological signals. As shown in Figure 5D and Note S6, electrical stimulation is delivered to the median nerve, after which electrodes are placed on the brain surface to serve as recording, reference, and ground electrodes. At a stimulation current of 20 mA, SSEPs from both upper and lower limbs (USSEP and LSSEP, respectively) are obtained following multiple trials of signal averaging and waveform superimposition. The waveforms reveal distinct N20/P25 for upper limbs, while lower limbs exhibit reproducible P37/N45 waveforms with prolonged latency (Figure 5E). Compared with conventional cup electrodes, the TDH electrodes demonstrate significantly enhanced amplitude in characteristic neurophysiological peaks, highlighting their ability to acquire higher‐fidelity neural signals. Statistical analysis of SSEP amplitude and latency reveals that TDH electrodes yield dramatically higher signal amplitudes than cup electrodes. Furthermore, SSEPs recorded from the lower limbs exhibit prolonged latencies, consistent with longer conduction pathways and greater signal attenuation compared to upper limb responses (Figure 5F).

### 2.6 Clinical Studies of Neurophysiological Monitoring

**FIGURE 6:**
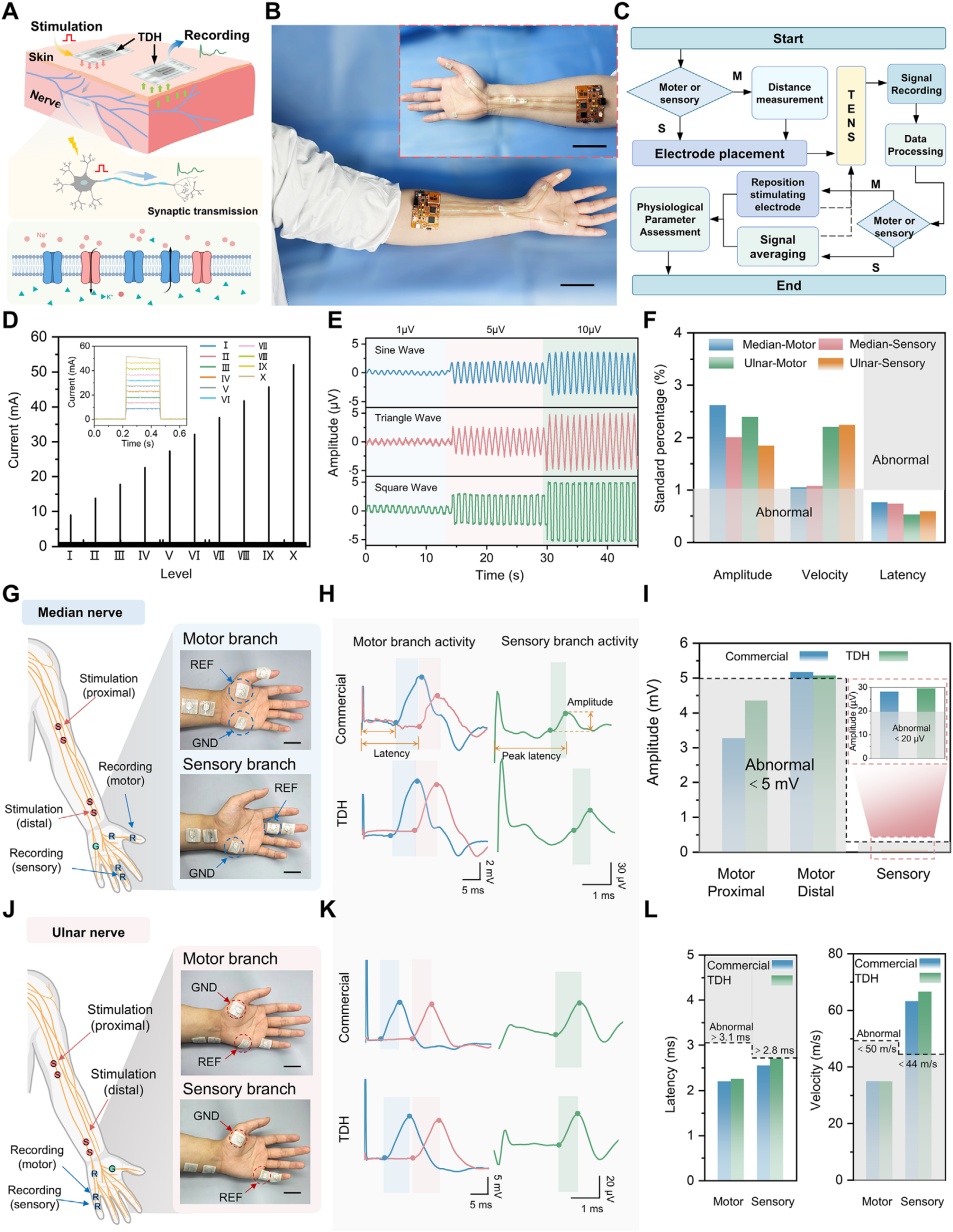
Clinical evaluation of neurophysiological function in sensory and motor nerve branches. (A) Schematic diagram of electromyographic signal conduction during stimulation and recording processes, including TDH/skin interface, neural pathways, and synaptic transmission. (B) Photograph of the TDH electronic system for neurophysiological signal acquisition in a healthy subject. (C) Standardized diagnostic procedures for assessing motor and sensory nerve branches. (D) Stimulation output characteristics of the TDH electronic system. (E) Sine wave, triangle wave, and square wave of different amplitudes received from the TDH electronic system. (F) The electrophysiological signals (motor and sensory) of the ulnar nerve and median nerve in a healthy subject. The threshold values of the amplitude, latency, and velocity are also displayed in the gray area. (G–I) Monitoring of the median nerve. Schematic of the electrode locations on the median nerve (G, left). Photographs of the TDH electrodes conformally attached to the skin for monitoring potentials from motor and sensory nerve branches (G, right). Corresponding neurophysiological activity recorded by commercial electrodes and TDH electrodes (H). Corresponding amplitudes of electromyography with the above‐mentioned electrodes (I). (J) Monitoring of the ulnar nerve. Schematic of the electrode locations on the ulnar nerve (J, left) and photos of the TDH electrodes attached to the hand of a subject for obtaining the motor and sensory signals of the ulnar nerve (J, right). (K) Comparison of the neurophysiological signals recorded by commercial and TDH electrodes in Case 4. (L) Comparison of latency and conduction velocity of the ulnar nerve using the abovementioned electrodes. Scale bars, 6 cm (B), 4 cm (G, J).

We developed a hybrid electronic system for neurophysiological monitoring and integrated TDH electrodes into the neurodiagnostic platform. Figure 6A shows the underlying mechanisms of neural conduction pathways. The hydrogel electrodes are attached to corresponding monitoring sites to ensure stable signal acquisition. When voltage pulses are applied to the stimulating electrodes, the action potentials are elicited in neurons via sodium ion (Na^+^) influx through voltage‐gated channels. The resulting electromyography signals propagate along neural pathways, subsequently acquired by the recording electrodes. The system transmits stimulation signals to the electrodes and acquires resulting electrophysiological data, which is processed, visualized, and stored in software (Figure 6B, Figure S22 and Note S7).

As shown in Figure 6C, the diagnostic workflow of the system begins with the operator selecting either motor or sensory branch measurement. For motor branch assessment, the nerve conduction velocity (NCV) is calculated based on the distance between distal and proximal electrode placements [50]. After positioning the electrodes, the transcutaneous electrical nerve stimulation module delivers current while the recording electrodes capture the signals. Afterwards, the stimulating electrode is moved to the distal site to repeat stimulation and recording. Finally, acquired signals are processed and analyzed to extract clinical parameters (Figure S23). Owing to the low signal intensity of sensory branches, ten cycles of signal superposition and averaging were required to achieve a sufficient SNR for analysis. Furthermore, the system must accommodate a wide dynamic range between stimulation and acquisition signals: the stimulation circuitry must stably deliver currents across varying amplitudes, while the acquisition front‐end must resolve signals ranging from microvolts (sensory region) to millivolts (motor branch). To validate performance under these constraints, we characterized the stability and acquisition fidelity of the system (Figure S24, Note S8). As shown in Figure 6D, the system is programmed to output ten distinct levels of stimulation current, ranging from 8 to 50 mA (8, 13, 18, 23, 27, 32, 36, 41, 46, and 50 mA) at a pulse duration of 0.25 s. An enlarged view confirms the stability of the current output across all levels. Waveforms ranging from low‐amplitude (1–10 µV) to high‐amplitude (20 mV) signals are stably recorded (Figure 6E and Figure S25). A 23‐year‐old healthy subject was tested to evaluate median and ulnar nerve function using the system (Figure 6F, Notes S9 and S10). The results show that the amplitude, latency, and velocity fall within normal ranges (i.e., motor branch: latency ≤ 4 ms, amplitude ≥ 5 mV, conduction velocity ≥ 50 m/s; sensory branch: latency ≤ 3.5 ms, amplitude ≥ 20 µV, conduction velocity ≥ 44 m/s).

To validate the diagnostic capability of the system for nerve injury, we conducted a series of tests in clinical cases. As shown in Figure 6G, the TDH electrodes are distributed over the sensory and motor branches of the median nerve in the hand of a subject to capture signals from each branch, respectively. In Case 1, a 51‐year‐old woman felt intermittent numbness in her thumb, as well as paresthesia and hypoesthesia in the hand and wrist, accompanied by persistent finger pain and numbness. As shown in Figure 6H, results obtained by the hydrogel electrodes demonstrate that the motor distal latency is prolonged to 5.21 ms and the proximal amplitude is reduced to 3.27 mV, compared with normal values (≤ 4 ms and ≥ 5 mV, respectively) (Figure 6I andFigure S26 and Table S2). Furthermore, all sensory branch parameters remain within normal limits (latency ≤ 3.5 ms, amplitude ≥ 20 µV, conduction velocity ≥ 44 m/s). Thereby, these findings are correlated with motor branch dysfunction, which indicates the occurrence of carpal tunnel syndrome or abductor pollicis brevis atrophy.

An examination of the median nerve was performed on a male patient who presented with anterior wrist pain, numbness, weakness in the middle finger, and hypoesthesia in the hand and wrist (Case 2). Given the relatively slack skin of the patient, the TDH electrodes achieved superior conformal adhesion. Consequently, at the proximal site, the TDH electrodes record a higher signal amplitude than the commercial electrodes, yielding more accurate results. In contrast to commercial electrodes, which lead to erroneous diagnosis (4.61 mV, normal threshold: ≥ 5 mV), the TDH electrode maintains conformal adhesion to slack or hairy skin surfaces (6.2 mV ≥ 5 mV), thereby enhancing signal acquisition fidelity and reducing diagnostic inaccuracies. The sensory amplitude is significantly reduced to 9.4 µV (normal values > 20 µV), and the latency is prolonged to 5.0 ms (normal values < 3.5 ms). Additionally, the conduction velocity is slowed to 34.6 m/s in the affected limb, compared with the normal value (> 44 m/s). Collectively, the results obtained with the TDH electrodes demonstrate significant impairment of the sensory branch with preserved motor branch, accounting for the observed symptoms (Figure S27, Table S3). Additionally, we conducted a comparative examination of the median nerves in both limbs of the patient. In Case 3, a 26‐year‐old female reported bilateral numbness and pain upon gripping objects. The electrophysiological findings are as follows: for the left limb, the sensory branch amplitude is reduced to 10.8 µV with a prolonged latency of 4.7 ms, the motor distal latency is significantly prolonged at 5.7 ms; for the right limb, only the motor distal latency is mildly prolonged at 4.2 ms (normal: < 3.5 ms). These results confirm the presence of bilateral carpal tunnel syndrome, with more severe impairment on the left side (Figure S28, Table S4).

We further evaluated the performance of the system in assessing ulnar nerve injury (Figure 6J). TDH electrodes were precisely positioned over both the sensory and motor branches of the ulnar nerve to capture signals from each branch for comprehensive assessment. A 26‐year‐old man underwent surgery on his left hand, and skin breakdown was observed postoperatively (Case 4). We performed ulnar nerve electromyography using both hydrogel and commercial electrodes (Figure 6K). According to results acquired from two types of electrodes, the conduction velocity of the motor branch of the ulnar nerve is slightly decreased (35 m/s, normal ≥ 44 m/s). The findings indicate that the sensory branch of the ulnar nerve is normal, whereas the motor branch of the radial nerve is impaired (Figure 6L, Figure S29, and Table S5). Due to the presence of skin wounds, the removal of the commercial electrode must be performed slowly and carefully to prevent secondary injury, causing discomfort and resulting in highly inefficient detachment. In contrast, the TDH electrode enables gentle, on‐demand detachment through controlled heater activation, ensuring the complete avoidance of skin damage. Another 59‐year‐old man (Case 5) presented with hypoesthesia in its sensory distribution, alongside muscle weakness and atrophy in its motor distribution. The sensory nerve action potential amplitude is reduced to < 12 µV (normal: > 17 µV). In the motor branch, the distal latency is prolonged to > 4 ms (normal: < 3.1 ms), and the conduction velocity is slowed to < 40 m/s (normal: > 50 m/s). Additionally, both the proximal and distal motor amplitudes are markedly reduced (proximal: 0.5 mV; distal: 0.28 mV). The results reveal abnormalities in the right ulnar nerve, indicating probable serious damage to the motor branch (Figure S30 and Table S6). During the removal process, the adhesion of the TDH electrode was reduced through thermal activation, enabling autonomous detachment and ensuring patient comfort.

In summary, the excellent flexibility and strong adhesion of the hybrid electronic system enable high‐fidelity electromyography monitoring on curvy skin surfaces, including highly curved fingers and slack skin in the elderly. Furthermore, its thermoresponsive properties prevent secondary damage at the wound site, thereby significantly improving patient comfort.

## 3 Conclusion

In this study, we have reported a printed hydrogel hybrid electronic system with thermoresponsive adhesion for neurophysiological monitoring. This integrated system consists of hydrogel electrodes, custom‐designed signal acquisition hardware, and analytical software. We first synthesized NIPAM‐based thermosensitive microspheres (THMs) via inverse emulsion polymerization. By tuning the rheological properties of the precursor solution, we achieved on‐demand and gradient control of the ink viscosity to facilitate direct printing. Furthermore, based on a grafting strategy, we synthesized CMC‐DA to improve the initial adhesion of hydrogels through multimodal chemical and physical interactions. The tensile test verified that the grafted layer significantly enhanced the interfacial adhesion strength (peel strength: 8.215 kPa, elastic modulus: 1.275 kPa, fracture strain: 223.264%). It is noted that the TDH achieves wide‐range adhesion regulation through a hydration‐dehydration strategy of hydrogel microspheres triggered by heat, facilitating rapid and tight coupling with skin for high‐quality signal monitoring and gentle detachment without causing skin injury. A comparative analysis is conducted between TDH and current hydrogel interfaces employed in flexible electronics (Table S1). TDH exhibits superior characteristics, including tunable adhesion based on water absorption/desorption mechanisms of hydrogel microspheres and ultra‐low modulus, outperforming existing hydrogel interfaces, particularly in terms of direct printability. The printable microsphere‐based inks enable rapid, high‐precision patterning of hydrogel interface layers, dramatically enhancing manufacturing efficiency for hydrogel electronic systems. TDHs can also be utilized as universal interface layers and achieve seamless integration with various tissues and soft substrates, demonstrating considerable potential for on‐skin devices/systems in bioelectronics. The TDHs maintain robust adhesion to complex skin surfaces (e.g., hairy skin and slack skin in the elderly), facilitating the acquisition of high‐fidelity and low‐artifact raw data. Furthermore, their thermoresponsive properties enable on‐demand detachment via thermal activation, thereby protecting the delicate skin by preventing secondary injury during removal from the wound site. In addition, the hydrogel interface layer developed in this work contains hydrophilic polymer segments (e.g., PSBMA chains), allowing polar water molecules from sweat to permeate into the polymer network and induce slight swelling due to water absorption. Owing to the dense crosslinked network, the swelling degree is constrained to a low level. Notably, during the adhesive tests and electromyography measurements on sweaty skin, the hydrogel device maintains superior adhesion and high SNR, further confirming the stability of hydrogel interface layers in sweat conditions.

By optimizing adhesion, softness, and electrical conductivity, the on‐skin electrode achieves significantly enhanced stimulation efficiency and signal recording quality. Compared with commercial electrodes, the TDH electrodes demonstrate lower contact impedance, higher SNR, and superior CIC and CSC values. Furthermore, due to the modification of TDH interface layers, the hydrogel electrodes enable high‐quality signal recording, even under complicated conditions (e.g., wet skin or under vibration). Through analysis of the electromyographic signal, the system enables accurate diagnosis of various peripheral neuropathies, including those affecting distinct nerves (median and ulnar) and different neural branches (motor and sensory). Clinical trials validate that the system enables more accurate diagnosis compared with the commercial device. The integrated system and a wireless design enable portable measurements and offer improved stimulation and signal acquisition performances. As a result, the soft hybrid electronic system shows potential for applications in a wider range of neurological disorder monitoring scenarios, including various nerve types and locations, while ensuring patient comfort and operational convenience during monitoring.

## 4 Experimental Section

### 4.1 Materials

All chemicals were used without further purification unless otherwise mentioned. NIPAM was obtained from TCI Shanghai Chemical Industry Development Co., LTD. Span‐80, benzophenone, DI water, potassium persulfate (KPS), N,N'‐Methylenebi‐s(acrylamide) (MBAA), α‐ketoglutaric acid (α‐KGA), poly (ethylene glycol) diacrylate (PEGDA), ascorbic acid (AA), 1‐(3‐Dimethylaminopropyl)‐3‐ethylcarbodiimide hydr‐ochloride (EDC), sodium hydroxide (NaOH), 4‐morpholineethanesulfonic acid (MES), N‐Hydroxysuccinimide (NHS) were obtained from Aladdin. Polyaspartic acid (PASP, Mw = 7000–8000), Acrylamide (AAm), Dopamine hydrochloride (DA) were obtained from Macklin. Cyclohexane (CYH) was purchased from Chengdu Kelong Chemical Co., Ltd. [2‐(methacryloyloxy) ethyl] dimethyl‐(3‐sulfopropyl) (SBMA) was purchased from Shanghai Yuanye Bio‐Technology Co., Ltd. Carboxymethylcellulose sodium (CMC) was purchased from Shanghai Bide Pharmatech Ltd. All pig skin for adhesion experiments was purchased from Yuzhixuan Frozen Food.

### 4.2 Preparation of MES Buffer Solution

MES buffer solution was prepared by dissolving MES (4 mmol) and NaCl (2 mmol) into 40 mL of DI water. Then, NaOH was added to the MES solution, adjusting the pH to 5.5.

### 4.3 Synthesis of Thermoresponsive PNPMs

First, 13.96 wt% NIPAM, 0.70 wt% PASP, and 0.11 wt% MBAA were dissolved in DI water. The solution was sonicated for 5 min at 25°C in a water bath. Subsequently, the solution was magnetic stirred at 1000 rpm for 5 min to ensure uniform dispersion to obtain the microgel pre‐polymerization solution. The solution was transferred into a separatory funnel. Then, a three‐necked flask containing 62.61 wt% cyclohexane and 1.40 wt% Span‐80 was placed in a 500 mL glass container. The DI water was poured into the glass container until the liquid level in the container equaled that in the three‐necked flask, followed by magnetic stirring at 1000 rpm for 10 min. Afterwards, the separatory funnel was placed in the middle neck of the three‐necked flask, and the stopcock of the separatory funnel was kept closed. The microgel pre‐polymerization solution was slowly added to the flask at 1 drop per second. The nitrogen gas was introduced into the upper end of the separatory funnel and allowed to slowly degass for 5 min. Subsequently, 0.28 wt% KPS was added to the mixture and magnetically stirred for 10 min. The polymerization was thermally initiated at 70°C for 3 h. Upon cooling to 20°C, the microspheres were washed with DI water and ethanol to obtain NIPAM‐PSAP microspheres (NPMs). Then, the NPMs were placed in a stainless petri dish, rapidly frozen using liquid nitrogen, and freeze‐dried (SJIA‐10N, Ningbo Shuangjia) at −35°C for 48 h, yielding the thermoresponsive PNPMs.

### 4.4 Synthesis of the TDH Ink

CMC (8 g) was dispersed in the MES buffer solution, then the solution was placed on a magnetic stirring hot plate and stirred at a speed of 200 rpm. After the solution was uniformly dispersed, 20 mmol each of EDC, NHS, and ascorbic acid were dissolved in the solution under continuous stirring. The mixture was subjected to 10 min of nitrogen degassing, yielding the CMC prepolymer solution. Then, 20 mmol DA was dissolved in 20 mL of MES solution. After the solution is uniformly dispersed, the CMC prepolymer solution was added to the DA‐MES solution and stirred for 12 h under a nitrogen atmosphere, yielding the CMC‐DA polymer solution. Subsequently, the CMC‐DA polymer solution was dialyzed against DI water for 24 h using a cellulose membrane (MD77: 1000 Da; Viskase, USA). Afterwards, the dialyzed CMC‐DA polymer solution was transferred into a stainless steel petri dish, rapidly frozen using liquid nitrogen, and freeze‐dried (SJIA‐10N, Ningbo Shuangjia) at −35°C for 48 h, yielding the grafted CMC‐DA. Subsequently, 8.17 wt% AAm, 8.17 wt% SBMA, and 0.54 wt% CMC‐DA were dissolved in 11 mL of DI water. Then, 0.35 wt% PEGDA and 1.09 wt% α‐KGA were added to the solution, followed by thorough stirring. Afterwards, 21.78 wt% THMs (with 75 µm sieve) were added to the solution. The mixture was subjected to nitrogen degassing for 10 min, followed by vacuum treatment in a vacuum reactor for 30 min, yielding the TDH ink.

### 4.5 Adhesion Evaluation of TDH Interface Layers

The standard tensile test (ASTM F2258) was employed to evaluate the adhesive strength of TDH interface layers. The TDH specimens were prepared with dimensions of 25 mm × 25 mm × 1 mm (length × width × thickness). Next, the top surface of hydrogels and the bottom surface of pigskins were bonded to a T‐shaped aluminum block by using cyanoacrylate adhesive. Then a preload of 10 N was applied to the surface for 10 s. Another heating plate was adhered to another T‐shaped aluminum block to heat the TDH. All tests were executed at a consistent peeling speed of 100 mm/min. The adhesion strength was calculated by dividing the maximum force by the contact area (6.25 cm^2^). Each strength value corresponded to the average of at least three experiment results. Deionized water was sprayed onto the interface to accelerate its rehydration rate after the hydrogel interface layer was dehydrated.

### 4.6 Rheological Measurements

DHR rheometer (TA Instruments, Discovery HR20, USA) was used to evaluate the performance of PNPMs. Before the test, the rheometer was equipped with a 60.0 mm parallel plate and a gap of 1.0 mm. The sample was prepared by dissolving 25% (w/w) of the different sizes PNPMs in DI water. Afterwards, the solution was subjected to nitrogen degassing for 5 and 30 min of vacuum treatment. In the oscillation mode, a sinusoidal amplitude sweep was carried out on different samples (250, 75, and 50 µm). The storage modulus (G′) and loss modulus (G″) were recorded at 25°C and 40°C within a strain range of 1%, at a frequency of 1 Hz for 300s. In the rheometer rotary tests, the viscosity‐temperature curves were measured with a shear rate sweep of 1–100 s^−1^. In the flow mode, viscosity‐shear rate and stress‐shear rate tests were performed under 20°C with a shear rate of 1–1000 s^−^^1^. Additionally, to further evaluate the thixotropic behavior of PNPMs, viscosity curves were measured under different shear strain intervals (3ITT). A shear frequency of 1 Hz was used, with a constant shear rate of 1 s^−^^1^ applied for 40 s, followed by a high shear rate of 1000 s^−^^1^ for 20 s. Subsequently, the shear rate was recovered to 1 s^−^^1^ and maintained for 40 s.

### 4.7 Process of Direct Printing

A high‐precision direct printing platform (customized model 101, Guangdong Sigu Technology Co., Ltd.) was used to prepare the TDH interface layer. The ink was filled into the material tube of the direct printing platform, and printing was conducted on a glass substrate. The air pressure, stepping speed, and nozzle height of the direct printing platform were adjusted appropriately, and direct writing was carried out in a serpentine pattern. Among them, the length of the segment was 25 mm, the length of the segment was 1.5 mm, with a total of 16 rows. After the completion of direct printing, the substrate was transferred to an ultraviolet lamp for 10 min of 300 W ultraviolet irradiation.

### 4.8 Materials Characterizations

Scanning electron microscopy (SEM, Hitachi SU3900) was used to characterize the surface morphology of samples. The presence of catechol groups was confirmed using a UV–vis spectrophotometer (Lambda 35, PerkinElmer, USA). The chemical composition of TDHs was analyzed using a Fourier‐transform infrared (FTIR) spectrometer (Nicolet iS50, Thermo Fisher) equipped with a germanium attenuated total reflection (ATR) crystal (55°C) and X‐ray photoelectron spectroscopy (XPS) (AXIS‐ULTRA DLD‐600 W, Kratos). The structural characterization of the synthesized CMC‐DA was performed using a proton nuclear magnetic resonance (^1^H NMR) spectrometer (Ascend 600 MHz, Bruker) in deuterated water (D_2_O), and subsequent spectral analysis was conducted using MestReNova software.

### 4.9 Characterizations of Stimulation and Recording Performances

To assess the recording performances of the TDH electrodes, an LCR meter (E4980A, Keysight) was used to record the contact impedance. Before the tests, the skin was cleaned with alcohol wipes, and two electrodes were laminated onto the forearm at 30 mm. TDH electrodes were used as the working electrode, while Ag/AgCl gel electrode was employed as the ground electrode. To simulate sweating conditions, artificial sweat was sprayed onto the skin. Then, the subject grasped the silicone ring, and the sEMG signals were obtained via data acquisition hardware (USB‐6218, National Instruments, USA). The waveforms were displayed on LABVIEW, and the data were analyzed using MATLAB (MathWorks, USA). The SNR of the sEMG signals was computed according to the analytical formulation provided in Note S3. The CIC and CSC were calculated via an electrochemical workstation (AUTOLAB, AUT85264). To evaluate the stimulation performance, the TDH electrode was used as the working electrode with an area of 1 cm^2^. A Pt electrode and Ag/AgCl electrode served as the counter electrode and reference electrode, respectively. All electrodes were immersed in PBS buffer solution, with the connecting wire separated from the liquid surface. In the CIC test, four cycles of ± 0.5 V pulse were applied with a pulse width of 0.1 s. The CSC was measured by integrating cyclic voltammetry curves. The scanning voltage was set to −0.6 to 0.8 V, with a scanning speed of 50 mV/s. According to the curve, CIC and CSC values were calculated by the formula provided in Note S4.

### 4.10 Biocompatibility Studies

The TDH samples were in high‐glucose Dulbecco's Modified Eagle's Medium (HG‐DMEM; Gibco, USA) for 24, 72, and 120 h. A blank maintained in HG‐DMEM alone served as the control group. HaCaT cells (iCell Bioscience Inc., RRID: CVCL_0038) were cultured in the abovementioned media at 37°C. Cellular viability was assessed via Calcein‐AM/PI dual fluorescence staining (Shanghai Yesen Biotechnology Co., Ltd., China). The relative cell viability and proliferation rates were quantitatively assessed using the CCK‐8 assay (Beyotime, Shanghai, China).

### 4.11 Animal Test Procedures

The animal study was approved by the Animal Care Committee of Huazhong University of Science and Technology under number IACUC 2023–3868. Adult male Japanese white rabbits (3.5 kg, Wuhan Wanqianjiaxing Biotechnology Co., Ltd.) were used in our studies. All rabbits were housed under a 12‐h light/dark cycle at 22°C–25°C conditions. Before the SSEP test, the animals were anesthetized intraperitoneally with pentobarbital sodium (30 mg/kg), and then the animals’ head fur was shaved. The TDH electrode was attached to the primary somatosensory cortex of the rabbits as the recording electrode. Two cup electrodes were attached to the frontal position, and the nasion position served as the reference and ground electrode, respectively. Two needle electrodes were inserted into the median nerve of the upper limb and the tibial nerve of the lower limb to provide stimulation, respectively.

### 4.12 Clinical Studies

The research protocol was approved by the ethics committee of Union Hospital, Tongji Medical College, Huazhong University of Science and Technology under number IEC 2025‐0280‐02. Six representative patients were reported in clinical studies. All patients were fully voluntary with informed consent. During the monitoring process, all patients were in a supine position without any extraneous movements. The electrodes and the system were applied to their skin by surgeons. For comparison, a commercial EMG/NCS/EP neurodiagnostic system (Natus Medical Incorporated) was simultaneously used to monitor and generate analysis reports. Postoperative judgments did not rely on the measurement results of the TDH electronic system or commercial devices; instead, diagnostic interventions were performed by experienced surgeons with more than 10 years of clinical experience.

### 4.13 Statistical Analysis

The experiment data were expressed as the mean ± SD unless otherwise specified. For statistical analysis, one‐way analysis of variance (ANOVA) with LSD post‐hoc test was performed using SPSS software (version 25.0, SPSS Inc.). All replicate numbers, error bars, P values, and statistical tests were indicated in figure legends with statistical significance at *p < 0.05, **p < 0.01, ***p < 0.001, or ****p < 0.0001.

## Author Contributions

H.W. conceived the project and designed the research; B.P. and G.Y. led the experiments and collected the overall data; G.Y. designed and fabricated the hydrogel materials; B.P. and G.Y. contributed to the characterizations; G.Y. designed the in vitro biocompatibility tests; B.P., G.Y., J. W., B.X., H.G., and T.B. designed the animal‐model studies; B.P., G.Y. and H.W. wrote the manuscript, and all authors contributed to reviewing and providing feedback on the manuscript. No AI‑assisted tools or machine‐generated content were used in the preparation of this work. All scientific content, data, and conclusions were fully reviewed, verified, and revised by the authors, who take full responsibility for the quality and originality of the manuscript.

## Conflicts of Interest

The authors declare no conflicts of interest.

## Supporting information




**Supporting File 1**: advs76396‐sup‐0001‐SuppMat.docx.

**Supporting File 2**: advs76396‐sup‐0002‐MovieS1.mp4.

## Data Availability

The data that support the plots of this study are available from the corresponding authors upon reasonable request.
