# Supplementary material for: A Printed Hydrogel Hybrid Electronic System With Thermoresponsive Adhesion for Neurophysiological Monitoring
Source: Adv Sci (Weinh). 2026 Jun 29;13(54):e76396. doi: 10.1002/advs.76396 (PMC13337101; doi:10.1002/advs.76396)
Supplement: Supplementary file 1 [file ADVS-13-e76396-s001.docx]

Supplementary information

**A printed hydrogel hybrid electronic system with thermoresponsive adhesion for neurophysiological monitoring**

Bo Pang^1†^, Ganguang Yang^1†^, Jiacheng Wen^1^, Hangyu Gong^1^, Caixin Gong^1^, Yuqi Qiu^1^, Zhixin Wang^1^, Qingyang Zheng^1^, Sen Zhou^1^, Tianzhao Bu^1^, Jia Tian^2^, Zhouping Yin^1^, Yutian Liu^2*^, Hao Wu^1,3*^

^1^Flexible Electronics Research Center, State Key Laboratory of Intelligent Manufacturing Equipment and Technology, School of Mechanical Science and Engineering, Huazhong University of Science and Technology, Wuhan 430074, China.

^2^Department of Hand Surgery, Union Hospital, Tongji Medical College, Huazhong University of Science and Technology, Wuhan, Hubei, 430022, China.

^3^School of Integrated Circuits, Huazhong University of Science and Technology, Wuhan, Hubei, 430074, China.

^*^Corresponding author. Email: hwu16@hust.edu.cn; [2016xh0053@hust.edu.cn](mailto:2016xh0053@hust.edu.cn)

**Table of Contents**

**Supplementary Notes**

**Note S1.** Design and integration of electrical components.

**Note S2.** Theoretical calculations of direct printing parameters.

**Note S3.** Calculations of peel energy.

**Note S4.** Calculations of SNR.

**Note S5.** Calculations of charge injection capacity and charge storage capacity.

**Note S6.** Analysis of SSEP signals.

**Note S7.** Design and integration of biosensors.

**Note S8.** Set up and characterizations of electrical stimulation.

**Note S9.** Calculations of conduction velocity.

**Note S10.** Neurophysiological data acquisition, processing, and analysis.

**Supplementary Figures**

**Fig. S1.** Synthesis processes of thermoresponsive directly-printed hydrogel microspheres.

**Fig. S2.** Characterizations of the thermoresponsive PNDMs.

**Fig. S3.** High-precision direct-printing hydrogel platform.

**Fig. S4.** The effects of direct printing parameters on line width (*l_line_*).

**Fig. S5.** Viscosity-shear rate and shear stress-shear rate curves of the thermosensitive ink and microspheres.

**Fig. S6.** Characterizations of the CMC-DA chains.

**Fig. S7.** Composition analysis of TDHs.

**Fig. S8.** Adhesion comparisons of different hydrogel interface layers.

**Fig. S9.** Adhesion energy of TDHs on various pig tissue substrates.

**Fig. S10.** Temperature-heat flow curves of TDH.

**Fig. S11.** The adhesion stability of TDHs.

**Fig. S12.** Tensile properties of TDHs.

**Fig. S13.** Mechanical performance characterizations of TDHs.

**Fig. S14.** Schematics of the preparation processes of the TDH electrode.

**Fig. S15.** Adhesive strength of TDHs when adhered to the pig skin at 20-40°C cycles.

**Fig. S16.** Thermal images and simulation before and after heating.

**Fig. S17.** Mechanical performances of the TDH electrode.

**Fig. S18.** Impedance and signal-to-noise ratio (SNR) of the TDH electrode at different temperatures.

**Fig. S19.** Charge injection curves (CIC) of different electrodes in 4 cycles.

**Fig. S20.** Biocompatibility evaluation of flexible epidermal electrodes.

**Fig. S21.** Long-term stability test under physiological conditions.

**Fig. S22.** Design of graphical user interface (GUI).

**Fig. S23.** Photographs of the TDH electrodes conformally attached to the proximal stimulation position of the arm.

**Fig. S24.** Layout of the flexible acquisition circuit board for neural electrophysiological examination.

**Fig. S25.** Recording signals using the TDH electronic system from a signal generator.

**Fig. S26.** Monitoring of neuropathy of the median nerve (Case 1).

**Fig. S27.** Monitoring of neuropathy of the median nerve (Case 2).

**Fig. S28.** Monitoring of neuropathy of the median nerve (Case 3).

**Fig. S29.** Monitoring of neuropathy of the ulnar nerve (Case 4).

**Fig. S30.** Monitoring of neuropathy of the ulnar nerve (Case 5).

**Supplementary Tables**

**Table S1.** Comparisons of reported hydrogel interface layers for flexible electronics.

**Table S2.** Comparisons of commercial electrodes and TDH electrodes for median nerve injury diagnosis (Case 1).

**Table S3.** Comparisons of commercial electrodes and TDH electrodes for median nerve injury diagnosis (Case 2).

**Table S4.** Comparisons of commercial electrodes and TDH electrodes for median nerve injury diagnosis (Case 3).

**Table S5.** Comparisons of commercial electrodes and TDH electrodes for ulnar nerve injury diagnosis (Case 4).

**Table S6.** Comparisons of commercial electrodes and TDH electrodes for ulnar nerve injury diagnosis (Case 5).

**Supplementary Movie**

**Movie S1.** Process of directly-printed hydrogel.

**Supplementary Notes**

**Note S1. Design and integration of electrical components.**

The biosensor components consist of five electrodes: stimulation anode electrode, cathode electrode, recording electrode, reference electrode, and ground electrode. All five biosensors were connected to the flexible acquisition front-end using anisotropic conductive film (ACF) and transparent flexible printed circuit boards (FPCB). The flexible printed circuit board for signal acquisition and transmission was custom-designed. The MCU (STM32F103RCT6, STMicroelectronics) was used to control the logical sequence of the entire system. The WIFI chip module (ALK8266WIFI) was used for wireless communication and signal transmission. The chip TENS-NS4 (Nanochap) provided a stimulation current. An analog front-end (ADS1299-6, Texas Instruments) was employed for data collection, which was connected to the MCU via the SPI protocol. A rechargeable Li-Po battery was employed to supply 3.7 V to the entire system. The power supply was regulated via TPS73633DBVR (Texas Instruments), providing a stable 3.3 V output for the system. Meanwhile, it supplied a 5.3 V reference voltage for the stimulator via TLV61048DBV (Texas Instruments), and a 5 V voltage for the AFE via TPS73250DBVR (Texas Instruments).

**Note S2. Theoretical calculations of direct printing parameters.**

In this study, a high-precision direct printing hydrogel platform was employed to characterize printing performance and fabricate the interface layer (Fig. S3). The flow rate through the nozzle is defined by the sum of the volume deposited per unit time and the volume extruded but not yet deposited. Assuming that the material deposited per unit time forms a rectangular prism with a width of *l_line_* (line width), a height of *h_line_* (line height), and a length equal to the platform stepping speed *V_line_*. For the material ejected from the nozzle but not yet reaching the direct printing platform per unit time, it is assumed to be cylindrical. The length corresponds to the distance from the nozzle to the direct printing platform (denoted as *h_nozzle_*), and its radius is half of the nozzle inner diameter *d_nozzle_*.

$$V_{line}l_{line}h_{line}+h_{nozzle}\frac{\pi d_{nozzle}^{2}}{4}=\frac{n}{3n+1}\left( \frac{\Delta p}{2\eta L} \right)\pi\left( \frac{d_{nozzle}}{2} \right)^{\frac{3n+1}{n}}$$

Here, *n* represents the flow index, *ΔP* denotes the pressure difference between the inlet and the nozzle, *η* is the viscosity, and *L* is the length of the print head. The parameter *n* is an intrinsic property of the material and is temperature-dependent.

We systematically investigated the effects of the applied pneumatic pressure (*ΔP*), the platform stepping speed (*V_line_*), and the nozzle-to-platform distance (*h_line_*) on the printed line width (*l_line_*). The line width was recorded under various parameter combinations during the tests. Through parameter optimization, the following optimal direct-printing parameters were determined (*ΔP*: 35-45 kPa, *V_line_*: 20-30 mm/s, *h_line_*: 0.25-0.35 mm).

**Note S3. Calculations of peel energy.**

Peel energy is defined as the integral of peel force with respect to displacement during the peeling process, as derived from the peel force-displacement curve, calculated by the contact area between the test sample and the porcine skin substrate. That is,

$$E_{tensile}=\frac{\int_{peeling\_start}^{peeling\_end} F_{x}dx}{A}$$

where *F_x_* denotes the real-time peel force, *dx* represents an infinitesimal displacement increment, and *A* is the contact area (taken as 6.25 cm² in this study).

**Note S4. Calculations of SNR.**

The signal-to-noise ratio (SNR) of the sEMG signals acquired by the electrodes was calculated using the following formula:

$$SNR(dB)=10{\times log}_{10} \frac{\sum_{k=1}^{N} V_{signal(k)}^{2}}{\sum_{k=1}^{N} V_{noise(k)}^{2}}$$

where *N* is the total number of recording samples. *V_signal_* and *V_noise_* denote the voltage amplitude of the signal and noise at the sample, respectively.

**Note S5. Calculations of charge injection capacity and charge storage capacity.**

Based on the charge injection curve, the charge injection capacity (CIC) was calculated as follows:

$$CIC=\frac{Q_{cathode}+Q_{anode}}{A_{test}}$$

where *Q_cathode_*, and *Q_anode_* denote the charge during the cathodal and anodal phases, respectively. *A_test_* represents the area of the test electrode.

The CSC value was obtained by integrating cyclic voltammetry curves using an electrochemical workstation. The value of CSC was calculated as follows:

$$CSC=\frac{\int\left| I \right|dt}{A_{test}}$$

where *I* and *A_test_* are the current and area of the test electrode, respectively.

**Note S6. Analysis of SSEP signals.**

We measured the somatosensory evoked potentials (SSEPs) in the upper and lower limbs of rabbits. Stimulation electrodes were positioned over the median nerve (upper limb) and the tibial nerve (lower limb), respectively. Under a stimulation current of 20 mA, reproducible waveforms were consistently recorded at the C4′ and Cz′ locations on the rabbit’s scalp. For the upper limb, a peak-to-valley potential was observed at approximately 20 ms and 25 ms, corresponding to the N20 (negative peak at 20 ms) and P25 (positive peak at 25 ms) components, respectively. For the lower limb, a valley-to-peak potential was identified approximately 37 ms and 45 ms, reflecting the P37 (positive peak at 37 ms) and N45 (negative peak at 45 ms) components. The amplitude was defined as the voltage difference between the peak and the valley.

**Note S7. Design and integration of biosensors.**

The PDMS (Dow Corning Sylgard 184) served as the substrate layer of the biosensor by 10:1 mixed, spin coating at 300 rpm for 30 s, and curing at 90 ℃ for 2h. Then, the wallpaper was attached to the PDMS as a mask and cut using a high-power picosecond ultraviolet laser precision machining platform (Jiangsu Rayto Laser Technology Co. Ltd., RA-EUV-L) at a power of 70% and speed of 200mm/s. Next, the ECCs were prepared according to the previous works, filled into the pre-cut patterns, and scraped flat using a blade. ^[1, 2]^ The ECC was cured at 160°C for 1.5h in an oven, and treated on the other side in the same way to obtain the electrode and heater layer. Subsequently, the electrode layer was treated with benzophenone. Finally, the hydrogel ink was printed onto the electrode and cross-linked by UV irradiation.

**Note S8. Set up and characterizations of electrical stimulation.**

The stimulation current of the electronic system was calculated via an oscilloscope. The anode and cathode of the stimulator were connected to a voltage resistor. The EEG signal simulator (H2-8000, Ming Sheng Electronic Technology Co., Ltd.) was used to verify the performance of the system in detecting tiny signals. The simulator generated standard sine, triangular, and square 1 Hz frequency signals with amplitudes of 1 μV, 5 μV, and 10 μV, respectively. The TDH electrodes were connected to the simulator's probe. Additionally, to simulate the dynamic range of EMG signals, a signal generator was used to generate the above three types of waveforms with an amplitude of 20mV. The same tests abovementioned were conducted, and the acquired signals were recorded.

**Note S9. Calculations of conduction velocity.**

Nerve conduction velocity (NCV) serves as an indicator of the physiological condition of nerve fibers within a nerve trunk and is defined as the ratio of conduction distance to conduction time. Specifically, sensory nerve conduction velocity is calculated using the distance between the stimulation and recording sites divided by the sensory latency.

However, motor nerve conduction velocity cannot be calculated using the same formula as sensory conduction due to the involvement of muscle. To eliminate the influence of neuromuscular transmission time, a two-site stimulation method (proximal and distal) is employed. Motor nerve conduction velocity is calculated by dividing the distance between the proximal and distal stimulation sites by the difference in latency between the two responses.

**Note S10. Neurophysiological data acquisition, processing, and analysis.**

The TDH electrodes were conformally attached to the skin. After a pulse signal stimulation, the PCB collected the signal and transferred it to the computer via Wi-Fi. The raw data of the motor branch could be analyzed directly, while the waveform of the sensory branch needed to be superposed and averaged. MATLAB (MathWorks, USA) with R2023b was used to analyze the signal. To obtain features with high fidelity, after performing band-pass filtering (0.5-10 Hz), the Signal Processing Toolbox 23.2 was used for visual playback of the data. We also measured the amplitude, conduction velocity, and latency of signals, which were commonly used as evaluation indicators.

**Supplementary Figures**

**Fig. S1. Synthesis processes of thermoresponsive directly-printed hydrogel microspheres.** (**A** to **C**) Synthesis of microsphere prepolymer solution via inverse emulsion polymerization. (**D** to **E**) Initiation of inverse emulsion polymerization (**F** to **I**), Centrifugal washing, and LT treatment to yield porous hydrogel microspheres. Scale bar, 80 μm.

**Fig. S2. Characterizations of the thermoresponsive PNDMs.** (**A**) Time-dependent changes in storage (G') and loss (G”) moduli of three microsphere solutions at 40°C. (**B**) Angular velocity dependence of storage (G') and loss (G″) moduli for three microsphere sizes (50 μm, 75 μm, 250 μm).

**Fig. S3. High-precision direct printing platform.** The system consists of two cameras: the positioning camera is used to determine the printing position, and the other observation camera is used to monitor the state of the nozzle. Hydrogel ink is pneumatically extruded through a micro-scale nozzle. The platform achieves the fabrication of TDH layers by precisely regulating height, applied air pressure, and printing speed. Scale bar, 0.5 mm.

**Fig. S4. The effects of direct printing parameters on line width (*l_line_*).** The parameters include the applied pneumatic pressure (*ΔP*), the platform stepping speed (*V_line_*), and the nozzle-to-platform heights (*h_line_*). (i)At a constant *V_line_* (25 mm/s) and *h_line_* (0.3 mm), the printed line width varies with *ΔP*. (ii) At a constant *ΔP* (40 kPa) and *h_line_* (0.3 mm), the printed line width varies with *V_line_*. (iii) At a constant *V_line_* (25 mm/s) and *ΔP* (40 kPa), the printed line width varies with *h_line_*.

**Fig. S5. Viscosity-shear rate and shear stress-shear rate curves of the** **thermosensitive ink and microspheres.**

**Fig. S6.** **Characterizations of the CMC-DA chains.** (**A**) UV-vis spectra of CMC and CMC-DA. (**B**) ¹H NMR spectra of CMC-DA and CMC.

**Fig. S7. Composition analysis of TDHs.** (**A**) Structural formula of TDH components. (**B** and **C**) XPS general scan spectra (B) and magnified view (C) of TDHs.

**Fig. S8. Adhesion comparisons of different hydrogel interface layers.** These hydrogels include CMC-only hydrogels without PNIPAM microspheres, CMC-DA-only hydrogels without PNIPAM microspheres, PNIPAM-only hydrogels, and TDH.

**Fig. S9. Adhesion energy of TDHs on various pig tissue substrates.**

**Fig. S10. Temperature-heat flow curves of TDH.** Endo down: the downward peak represents an endothermic peak.

**Fig. S11. The adhesion stability of TDHs.** (**A**) Adhesive strength of TDH during 5-day adhesion. (**B**) Adhesive strength of TDHs when adhered to the wet skin at 40°C and 20°C.

**Fig. S12. Tensile properties of TDHs.** (**A** and **B**) Force-stress curves (A) and elastic modulus (B) of Ecoflex, PDMS, and TDH.

**Fig. S13. Mechanical performance characterizations of TDHs.** (**A**) Hysteresis of TDHs at 50% strain cycles. (**B**) Weight retention comparisons of TDHs before/after 24-hour storage at 25°C and 56% humidity.

**Fig. S14. Schematics of the preparation processes of the TDH electrode.** (**A**) Preparation of PDMS substrate. (**B-D**) Preparation process for the electrically conductive heating layer. (**E-H**) Preparation process for ECC electrode. (**I**) Treatment with benzophenone solution. (**J**) Direct printing of TDH on electrodes. (**K**) Crosslinking of TDH under UV irradiation. (L) Peeling off the TDH electrode from the glass substrate.

**Fig. S15. Adhesive strength of TDHs when adhered to the pig skin at 20-40°C cycles.**

**Fig. S16. Thermal images and simulation before and after heating. (A)** Thermal images at 0-10 s of heating. **(B)** Thermal simulation.

**Fig. S17. Mechanical performances of the TDH electrode. (A)** ΔR/R_0_ of the electrode under different tensile strains. **(B)** ΔR/R_0_ of the electrode after 200 cycles of 20% cyclic strain.

**Fig. S18. Impedance and signal-to-noise ratio (SNR) of the TDH electrode at different temperatures. (A)** Comparisons of contact impedance at different temperatures. Inset shows the corresponding contact impedance of each electrode at 1 kHz. **(B)** SNR values of the abovementioned electrodes under 20, 30, and 40 ℃.

**Fig. S19. Charge injection curves (CIC) of different electrodes in 4 cycles.**

**Fig. S20.** **Biocompatibility evaluation of flexible epidermal electrodes.** (**A**) Skin condition after 20 peeling cycles. (**B**) Skin status following 60-minute continuous attachment. Scale bar, 10 mm.

**Fig. S21. Long-term stability test under physiological conditions. (A)** Comparisons of contact impedance at the initial state and after 24- and 48-hour attachment. The inset shows the corresponding contact impedance at 1 kHz. **(B)** SNR values of the abovementioned electrodes at the initial state, after 24- and 48-hour attachment. **(C)** Skin conditions at the initial state, after 24- and 48-hour attachment.

**Fig. S22. Design of graphical user interface (GUI).** The GUI controls the stimulation switch and signal acquisition of the board. The analog front-end (AFE) uses a 16k sampling rate, and the system connects to the PCB via Wi-Fi. When the system starts acquiring data, the stimulator emits a stimulation signal every second, which is displayed on the host computer. Simultaneously, the computer records the captured waveforms. The window of waveforms can be adjusted using a ruler tool, and the recorded data is exported for medical analysis.

**Fig. S23.** **Photographs of the TDH electrodes conformally attached to the proximal stimulation position of the arm.** (**A**) Ulnar nerve. (**B**) Median nerve. Scale bar, 10 mm.

**Fig. S24.** **Layout of the flexible acquisition circuit board for neural electrophysiological examination.** (**A**)Top layer. (**B**) Bottom layer. (**C**) Middle layer. (**D**) Photograph of the PCB board. Scale bar, 10 mm (D).

**Fig. S25.** **Recording signals using the TDH electronic system from a signal generator.** (**A**) Schematic of evaluating the performance of the system. The signal generator generated standard sine, triangular, and square wave signals with amplitudes of 20 mV and a frequency of 1 Hz. The TDH was connected to the end of the generator, and the data was transmitted to the host computer via the PCB system using Wi-Fi protocol. (**B**) The signal of different waveforms received from the TDH electronic system.

**Fig. S26. Monitoring of neuropathy of the median nerve (Case 1).** (**A**) Comparison of latency and conduction velocity of commercial and TDH electrodes. (**B**) The electrophysiological signals of the sensory and motor branches in Case 1. The prolonged motor latency and reduced amplitude recorded via both electrodes within the median nerve motor region indicate motor branch dysfunction, consistent with carpal tunnel syndrome or abductor pollicis brevis atrophy.

**Fig. S27.** **Monitoring of neuropathy of the median nerve (Case 2).** (**A**) Comparison of the neurophysiological signals recorded by commercial and TDH electrodes. (**B** and **C**) Comparison of latency, conduction velocity (B), and amplitude (C) of the median nerve using the abovementioned electrodes. (**D**) The evaluations of the electrophysiological signals of the sensory and motor branches in case 2. The assessment pie chart reveals that it exhibits prolonged distal motor latency, mildly prolonged sensory latency, reduced potential amplitudes, and decreased conduction velocity, findings consistent with carpal tunnel syndrome.

**Fig. S28. Monitoring of neuropathy of the median nerve (Case 3).** (**A**) Comparison of the neurophysiological activity recorded by bilateral median nerves. (**B** and **C**) Comparison of latency, conduction velocity (B), and amplitude (C) of the motor and sensory branches. (**D**) The evaluations of electrophysiological signals in case 3. The sensory regions of the median nerve in both left and right limbs show mildly prolonged latency. The sensory region of the right hand exhibits abnormally slowed conduction, with a mild reduction in amplitude, suggesting the possibility of bilateral carpal tunnel lesions.

**Fig. S29. Monitoring of neuropathy of the ulnar nerve (Case 4).** (**A**) Comparison of amplitudes of electromyography with commercial and TDH electrodes. (**B**) The electrophysiological signals of the sensory and motor branches in case 4. Both abovementioned electrodes detected localized slowing of motor conduction velocity in the ulnar nerve, indicating focal demyelination of motor fibers.

**Fig. S30. Monitoring of neuropathy of the ulnar nerve (Case 5).** (**A**) Comparison of the neurophysiological signals recorded by commercial and TDH electrodes. (**B** and **C**) Comparison of latency, conduction velocity (B), and amplitude (C) of the ulnar nerve using the abovementioned electrodes. (**D**) The evaluations of the electrophysiological signals of the sensory and motor branches in case 5. In the ulnar motor nerve, both abovementioned electrodes detected greater than 50% amplitude reduction, prolonged latency, and significantly decreased conduction velocity, indicating conduction block at the elbow.

**Supplementary Tables**

**Table S1. Comparisons of reported hydrogel interface layers for flexible electronics.**

| Materials | Fabrication process | Responsive | Tunable adhesion | Softness  (modulus) | Application | Ref. |
| --- | --- | --- | --- | --- | --- | --- |
| PVA/PVP/PDA NPs | Mold pouring | No | No | 6.9 kPa | EEG recording | [3] |
| PVA/PAA-S-S-NHS | Mold pouring | Yes  (Chemical agent: Na_2_CO_3_/ GSH) | Yes | 20 kPa | Bioadhesives | [4] |
| PBAc-NHS/SA | Mold pouring | Yes  (Chemical agent: glucose) | Yes | 2 kPa | Bioadhesives | [5] |
| Lignin/glycerol/ AM/SBMA | Mold pouring | No | No | ~10 kpa | ECG/EMG recording | [6] |
| PAA/BSA/Alg-DA | Mold pouring | No | No | >100 kPa | Bioadhesives | [7] |
| NIH | Mold pouring | No | No | 1.5 kPa | ECG recording | [8] |
| CNF-DA/PAA | Mold pouring | Yes  (UV light) | Yes | N/A | TENG | [9] |
| PAAm-Alg/Chitosan | Mold pouring | Yes  (Ultrasound) | Yes | N/A | Bioadhesives | [10] |
| PAA-NHS multilayer | Mold pouring | No | No | 70 kPa | Surgical stapler | [11] |
| AHBH | Mold pouring | No | No | 6.9 kPa | EEG recording | [12] |
| DGMH | Mold pouring | Yes  (Electrical field) | Yes | 3.0 kPa | Perforating artery  locating | [13] |
| **TDH** | **Direct printing** | **Yes**  **(Hydration/dehydration of microspheres)** | **Yes** | **~1 kPa** | **Neurophysiological monitoring** | **This**  **work** |

|  | **Table S2. Comparisons of commercial electrodes and TDH electrodes for median nerve injury diagnosis (Case 1).** | | | | | | | | |
| --- | --- | --- | --- | --- | --- | --- | --- | --- | --- |
| Indicator | | | Motor branch conduction | | | | Sensory branch conduction | | |
| Group | | | Distal  latency  (ms) | Distal amplitude  (mV) | Proximal amplitude  (mV) | Conduction velocity  (m/s) | Latency  (ms) | Amplitude (μV) | Conduction velocity (m/s) |
| Median nerve | | Right  (Com.^a)^) | **5.06** | 5.16 | **3.27** | 55.66 | 2.55 | 28.1 | 58.08 |
|  |  | Right  (TDH.^b)^) | **5.21** | 5.08 | **4.35** | 55.22 | 3.12 | 29.6 | 51.92 |
|  |  | Normal | ≤4 | ≥5 | ≥5 | ≥50 | ≤3.5 | ≥20 | ≥44 |
|  | Com.^a)^, commercial electrodes. TDH.^b)^, hydrogel electrodes. | | | | | | | | |

|  | **Table S3. Comparisons of commercial electrodes and TDH electrodes for median nerve injury diagnosis (Case 2).** | | | | | | | | |
| --- | --- | --- | --- | --- | --- | --- | --- | --- | --- |
| Indicator | | | Motor branch conduction | | | | Sensory branch conduction | | |
| Group | | | Distal  latency  (ms) | Distal amplitude  (mV) | Proximal amplitude  (mV) | Conduction velocity  (m/s) | Latency  (ms) | Amplitude (μV) | Conduction velocity (m/s) |
| Median nerve | | Right  (Com.^a)^) | **5.5** | 6.76 | **4.61** | 51.4 | **5** | **3.28** | **34.6** |
|  |  | Right  (TDH.^b)^) | **5.25** | 7.39 | 6.2 | 52.9 | **4.18** | **9.4** | **37.1** |
|  |  | Normal | ≤4 | ≥5 | ≥5 | ≥50 | ≤3.5 | ≥20 | ≥44 |
|  | Com.^a)^, commercial electrodes. TDH.^b)^, hydrogel electrodes. | | | | | | | | |

|  | **Table S4. Comparisons of commercial electrodes and TDH electrodes for median nerve injury diagnosis (Case 3).** | | | | | | | | |
| --- | --- | --- | --- | --- | --- | --- | --- | --- | --- |
| Indicator | | | Motor branch conduction | | | | Sensory branch conduction | | |
| Group | | | Distal  latency  (ms) | Distal amplitude  (mV) | Proximal amplitude  (mV) | Conduction velocity  (m/s) | Latency  (ms) | Amplitude (μV) | Conduction velocity (m/s) |
| Median nerve | | Left  (TDH.) | **5.7** | 7 | 7 | 56.94 | **4.74** | **10.8** | 60.37 |
|  |  | Right  (TDH.) | **4.2** | 8.7 | 8.5 | 56.76 | 3.23 | 22.1 | 59 |
|  |  | Normal | ≤4 | ≥5 | ≥5 | ≥50 | ≤3.5 | ≥20 | ≥44 |

|  | **Table S5. Comparisons of commercial electrodes and TDH electrodes for ulnar nerve injury diagnosis (Case 4).** | | | | | | | | |
| --- | --- | --- | --- | --- | --- | --- | --- | --- | --- |
| Indicator | | | Motor branch conduction | | | | Sensory branch conduction | | |
| Group | | | Distal  latency  (ms) | Distal amplitude  (mV) | Proximal amplitude  (mV) | Conduction velocity  (m/s) | Latency  (ms) | Amplitude (μV) | Conduction velocity (m/s) |
| Ulnar nerve | | Left  (Com.^a)^) | 2.2 | 11.82 | 10.55 | **34.88** | 2.55 | 23.7 | 63.19 |
|  |  | Left  (TDH.^b)^) | 2.25 | 13.41 | 12.14 | **34.74** | 2.7 | 29.6 | 66.50 |
|  |  | Normal | ≤3.1 | ≥5 | ≥5 | ≥50 | ≤2.8 | ≥17 | ≥44 |
|  | Com.^a)^, commercial electrodes. TDH.^b)^, hydrogel electrodes. | | | | | | | | |

|  | **Table S6. Comparisons of commercial electrodes and TDH electrodes for ulnar nerve injury diagnosis (Case 5).** | | | | | | | | |
| --- | --- | --- | --- | --- | --- | --- | --- | --- | --- |
| Indicator | | | Motor branch conduction | | | | Sensory branch conduction | | |
| Group | | | Distal  latency  (ms) | Distal amplitude  (mV) | Proximal amplitude  (mV) | Conduction velocity  (m/s) | Latency  (ms) | Amplitude (μV) | Conduction velocity (m/s) |
| Ulnar nerve | | Right  (Com.^a)^) | **4.08** | **0.27** | **0.33** | **36.59** | 2.6 | **11.6** | 67.36 |
|  |  | Right  (TDH.^b)^) | **5.21** | **0.28** | **0.51** | **35.43** | 2.08 | **9.1** | 65.87 |
|  |  | Normal | ≤3.1 | ≥5 | ≥5 | ≥50 | ≤2.8 | ≥17 | ≥44 |
|  | Com.^a)^, commercial electrodes. TDH.^b)^, hydrogel electrodes. | | | | | | | | |

**References**

[1]	D. Wei, J. Guo, Y. Qiu, S. Liu, J. Mao, Y. Liu, Z. Chen, H. Wu, Z. Yin, *Natl. Sci. Rev*. **2022**, 9, nwac227.

[2] W. Guo, P. Zheng, X. Huang, H. Zhuo, Y. Wu, Z. Yin, Z. Li, H. Wu, *ACS Appl. Mater. Interfaces* **2019**, 11, 8567.

[3]	Q. Han, C. Zhang, T. Guo, Y. Tian, W. Song, J. Lei, Q. Li, A. Wang, M. Zhang, S. Bai, X. Yan, *Adv. Mater.* **2023**, 35, 2209606.

[4]	X. Chen, H. Yuk, J. Wu, C. S. Nabzdyk, X. Zhao, *Proc. Nat. Acad. Sci. U. S. A.* **2020**, 117, 15497.

[5]	Y. Xue, J. Zhang, X. Chen, J. Zhang, G. Chen, K. Zhang, J. Lin, C. Guo, J. Liu, *Adv. Funct. Mater.* **2021**, 31, 2106446.

[6]	O. Hu, M. Lu, M. Cai, J. Liu, X. Qiu, C. F. Guo, C. Y. Zhang, Y. Qian, *Adv. Mater.*, **2024**, 2407129.

[7]	B. Xue, J. Gu, L. Li, W. Yu, S. Yin, M. Qin, Q. Jiang, W. Wang, Y. Cao, *Nat. Commun.* **2021**, 12, 7156.

[8]	G. Yang, Z. Lan, H. Gong, J. Wen, B. Pang, Y. Qiu, Y. Zhang, W. Guo, T. Bu, B. Xie, H. Wu, *Adv. Funct. Mater.* **2025**, 35, 2417841.

[9]	L. Zhang, L. Chen, S. Wang, S. Wang, D. Wang, L. Yu, X. Xu, H. Liu, C. Chen, *Nat. Commun.* **2024**, 15, 3859.

[10]	Z. Ma, C. Bourquard, Q. Gao, S. Jiang, T. De Iure-Grimmel, R. Huo, X. Li, Z. He, Z. Yang, G. Yang, Y. Wang, E. Lam, Z.-h. Gao, O. Supponen, J. Li, *Science* **2022**, 377, 751.

[11]	S. J. Wu, H. Yuk, J. Wu, C. S. Nabzdyk, X. Zhao, *Adv. Mater.* **2021**, 33, 2007667.

[12]	G. Yang, K. Zhu, W. Guo, D. Wu, X. Quan, X. Huang, S. Liu, Y. Li, H. Fang, Y. Qiu, Q. Zheng, M. Zhu, J. Huang, Z. Zeng, Z. Yin, H. Wu, *Adv. Funct. Mater.* **2022**, 32, 2200457.

[13]	G. Yang, Y. Qiu, B. Pang, W. Guo, S. Liu, Q. Zheng, S. Zhou, J. Tian, W. Liu, B. Xie, T. Bu, C. Wu, Z. Yin, Y. Liu, H. Wu, *Sci. Adv.* **2025**, 11, eadw6166.
